# Crosstalk between Long-Term Sublethal Oxidative Stress and Detrimental Inflammation as Potential Drivers for Age-Related Retinal Degeneration

**DOI:** 10.3390/antiox10010025

**Published:** 2020-12-29

**Authors:** Lara Macchioni, Davide Chiasserini, Letizia Mezzasoma, Magdalena Davidescu, Pier Luigi Orvietani, Katia Fettucciari, Leonardo Salviati, Barbara Cellini, Ilaria Bellezza

**Affiliations:** 1 Department of Medicine and Surgery, Section of Physiology and Biochemistry, University of Perugia, Piazza dell’Università, 1, 06123 Perugia PG, Italy; lara.macchioni@unipg.it (L.M.); davide.chiasserini@unipg.it (D.C.); letizia.mezzasoma@unipg.it (L.M.); magdalena.davidescu@unipg.it (M.D.); pier.orvietani@unipg.it (P.L.O.); katia.fettucciari@unipg.it (K.F.); barbara.cellini@unipg.it (B.C.); 2 Clinical Genetics Unit, Department of Woman and Child Health, University of Padova, Via 8 Febbraio 1848, 2, 35122 Padova PD, Italy; leonardo.salviati@unipd.it

**Keywords:** retinal pigment epithelium, ARPE-19, age-related macular degeneration, oxidative stress, senescence, proteomics, mitochondria, inflammasome

## Abstract

Age-related retinal degenerations, including age-related macular degeneration (AMD), are caused by the loss of retinal pigmented epithelial (RPE) cells and photoreceptors. The pathogenesis of AMD, deeply linked to the aging process, also involves oxidative stress and inflammatory responses. However, the molecular mechanisms contributing to the shift from healthy aging to AMD are still poorly understood. Since RPE cells in the retina are chronically exposed to a pro-oxidant microenvironment throughout life, we simulated in vivo conditions by growing ARPE-19 cells in the presence of 10 μM H_2_O_2_ for several passages. This long-term oxidative insult induced senescence in ARPE-19 cells without affecting cell proliferation. Global proteomic analysis revealed a dysregulated expression in proteins involved in antioxidant response, mitochondrial homeostasis, and extracellular matrix organization. The analyses of mitochondrial functionality showed increased mitochondrial biogenesis and ATP generation and improved response to oxidative stress. The latter, however, was linked to nuclear factor-κB (NF-κB) rather than nuclear factor erythroid 2–related factor 2 (Nrf2) activation. NF-κB hyperactivation also resulted in increased pro-inflammatory cytokines expression and inflammasome activation. Moreover, in response to additional pro-inflammatory insults, senescent ARPE-19 cells underwent an exaggerated inflammatory reaction. Our results indicate senescence as an important link between chronic oxidative insult and detrimental chronic inflammation, with possible future repercussions for therapeutic interventions.

## 1. Introduction

Age-related macular degeneration (AMD) is the principal cause of blindness in western countries [1]. In 55- to 59-year-old European subjects, the prevalence of early and late AMD is 3.5% (95% confidence interval (CI) 2.1–5.0%) and 0.1% (95% CI 0.04–0.3%), respectively. These data indicate that by 2040 the number of individuals suffering from early AMD will range between 14.9 and 21.5 million [1]. Blindness is caused by the loss of retinal pigmented epithelial (RPE) cells and photoreceptors in large zones of the macula. RPE cells, a monolayer of post-mitotic polarized epithelial cells sandwiched between photoreceptors and the choroid, are devoted to photoreceptor health and function. In particular, RPE cells possess specialized functions including phagocytosis of photoreceptor outer segments, light absorption and vitamin A metabolism [2]. RPE also provide crucial activities for Bruch’s membrane optimal function, in particular RPE cells synthesize specific laminins that permit the adherence of Bruch’s membrane to RPE through interaction with RPE-expressed integrins [2,3]. RPE and Bruch’s membrane dysfunctions have been implicated in the pathogenesis of AMD [2,3,4].

There is still no effective therapy for AMD; however, the administration of an antioxidant and vitamin cocktails can decrease the risk of AMD progression by approximately 30% (AREDS formulation [5]). The main risk factors for the development of AMD are aging, cigarette smoking, high fat diet and light-induced photooxidative reactions [2], which share the capacity to increase reactive oxygen species (ROS) generation and promote oxidative stress. Therefore, oxidative injury to RPE has been suggested as a major culprit of AMD pathogenesis and progression [6].

In this context, it is important to consider that the retina is constantly subjected to pro-oxidant stimuli. ROS generation occurs whenever light interacts with endogenous chromophores in the ocular tissue and as a by-product of mitochondrial electron transport chain [6]. RPE cells are enriched in mitochondria essential for oxidative phosphorylation and aerobic ATP production [7]. This is in line with the fact that retina is one of the highest oxygen consuming tissues in the human body [8], implying that the retina deals with moderate/high levels of ROS throughout life. However, AMD is an age-associated disease, suggesting that the long-term exposure to oxidative stress, more than the acute exposure, can contribute to retinal dysfunction. In fact, early in life, the excess of ROS is usually buffered by a range of antioxidant defenses; whilst, during aging, ROS buffering activities are often compromised leading to the accumulation of oxidative damage which, in turn, may cause tissue dysfunction [3]. Nonetheless, the molecular mechanism linking healthy aging to AMD pathogenesis are still poorly understood.

A central role for oxidative stress in several pathophysiological events, including cell senescence process, has been shown [9]. Cell senescence represents a major contributor to aging and age-related diseases, including AMD [9]. Senescent cells show peculiar changes in intracellular organization and metabolism. They are characterized by morphological alterations due to cytoskeletal rearrangements; increased lysosomal mass, as evidenced by the increased activity of the acidic senescence-associated β-galactosidase (SA-β-Gal); and increased mitochondrial biogenesis and respiration [9]. Furthermore, senescent cells actively secrete chemokines, cytokines and growth factors, collectively known as senescence-associated secretory phenotype (SASP) [9]. SASP can be hypothesized as a key player in senescence-related AMD pathophysiology [10,11] due to the observation that inflammation plays a key role in the pathogenesis of AMD [2]. In the complex scenario of inflammation, a central role is played by the inflammasome, a multimolecular platform devoted to the recognition of damage-associated molecular patterns (DAMPS) and pathogen-associated molecular patterns (PAMPS). Inflammasome activation results in the maturation of the highly pro-inflammatory cytokine IL-1β which has been linked to the excessive inflammation associated to AMD [12]. The inflammasome is activated in the RPE of AMD patients. It has been proposed that drusen components, complement proteins and oxidative by-products are responsible for inflammasome activation in RPE [13].

In this work, we demonstrated that a long-term sublethal oxidative insult induces a senescent phenotype characterized by modifications in intracellular organization and mitochondrial metabolism in ARPE-19 cell line. By proteomic analyses we also showed that chronic oxidative insult alters integrin signalling and the organization of extracellular matrix in ARPE-19 cells. We also provided evidence that long-term sublethal oxidative insult causes the induction of an inflammatory phenotype, which is further augmented by both sterile and pathogen-associated inflammatory stimuli.

These data highlight the importance of the crosstalk between inflammation and oxidative stress in RPE cells and offer a new cellular model for the high-throughput study of therapeutic interventions.

## 2. Materials and Methods

### 2.1. Reagents

All reagents, unless otherwise stated, are from Merck (Darmstadt, Germany). ATP Bioluminescent Assay Kit, 7′-dichlorodihydrofluorescein diacetate (H_2_DCFDA), antimycin A, valinomycin, protease inhibitor cocktail, and phosphatase inhibitor cocktail were from Sigma Aldrich (St. Louis, MO, USA). 5,5′,6,6′-tetrachloro-1,1′,3,3′-tetraethylbenzimidazolylcarbocyanine iodide (JC-1) and MitoSOX™ were from Molecular Probes (Invitrogen, Carlsbad, CA, USA).

### 2.2. Cell Culture, Population Doubling and Cell Viability

**Figure 1:**
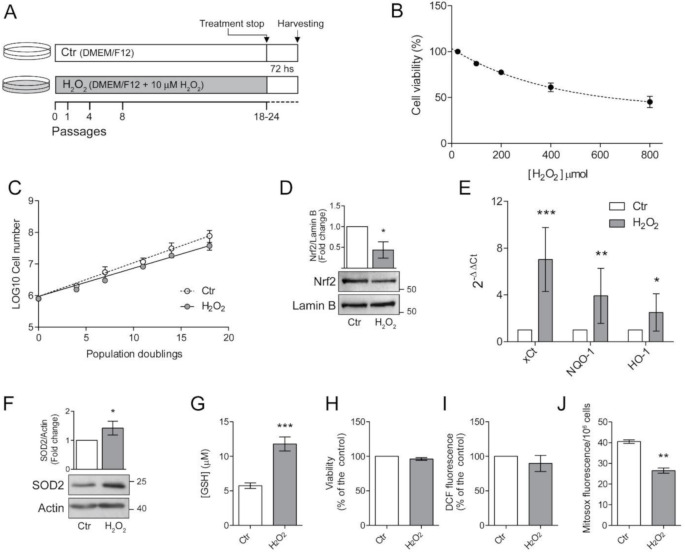
Chronic exposure to pro-oxidant insult increases antioxidant defences in ARPE-19 cells. (**A**) Schematic representation of the experimental model. ARPE-19 cells were grown for 18–24 passages in the presence of 10 μM H_2_O_2_ (H_2_O_2_) or left untreated (Ctr). Cells were seeded in H_2_O_2_-free medium and grown for 72 h before experiments; (**B**) ARPE-19 cell viability, determined by MTT assay, after exposure to increasing concentration of H_2_O_2_ (50–800 µM) for 48 h. Results are given as percentage of the control. Ctr and H_2_O_2_-treated cells were seeded and (**C**) cell growth was measured between p18 and p24 and reported as population doubling. Ctr and H_2_O_2_ treated cells were seeded in H_2_O_2_-free medium and grown for 72 h and used for (**D**) assessment of Nrf2 nuclear translocation by western blotting. Lamin B was used as loading control. Bar graphs represent the densitometric analysis reported as the ratio between Nrf2 and Lamin B band intensity. Untreated cells were used as control and assumed as 1. One representative blot is shown and densitometric analyses are expressed as the mean ± SD of three independent experiments; (**E**) analysis of NQO-1, xCt and HO-1 gene expression by qPCR. Gene expression values were normalized to GAPDH and reported as 2^−ΔΔCt^. Results were standardized to 1 in control samples. Data represent the mean ± SD from three independent experiments performed in triplicate; (**F**) analysis of SOD2 protein expression by Western blotting. β-actin was used as loading control. Bar graphs represent the densitometric analysis reported as the ratio between SOD2 protein and β-actin band intensity. Untreated cells were used as control and assumed as 1. One representative blot is shown and densitometric analyses are expressed as the mean ± SD of three independent experiments; (**G**) determination of GSH intracellular levels by DTNB assay. GSH level was analysed in 1 × 10^6^ cells and reported as μM. Bar graphs represent the mean ± SD of five independent experiments; (**H**) determination of cell viability by MTT assay. The absorbance of control cells was assumed as 1. Data represent the mean ± SD of three independent experiments; (**I**) measurement of total intracellular ROS levels by H_2_DCFDA staining (λ_ex_ = 485 nm; λ_em_ = 535 nm) normalized to cell viability. Fluorescence values in control cells was assumed as 1. Data represent the mean ± SD of three independent experiments; (**J**) measurement of mitochondrial ROS by MitoSox Red staining (λ_ex_ = 510 nm; λ_em_ = 600 nm). Data represent the mean ± SD of three independent experiments (* *p* < 0.05, ** *p* < 0.01, *** *p* < 0.001).

Retinal epithelial ARPE-19 cells (ATCC #CRL-2302) were cultured in DMEM/F12 supplemented with 10% Foetal bovine serum (FBS), penicillin (100 U/mL) and streptomycin (100 mg/mL) at 37 °C in a humidified incubator under 5% CO_2_. One batch of cells was routinely grown in the presence of 10 μM H_2_O_2_ for 24 passages. Medium was changed every 3–4 days and H_2_O_2_ was added at every medium change. Cells were passaged twice a week with a 1:3 sub-culture ratio. To avoid the detection of effects caused by acute H_2_O_2_ treatment, cells were seeded (30,000 cells/cm^2^) in H_2_O_2_-free medium, grown for 72 h and then used for the experiment (Figure 1A).

Growth rate was measured between p18 and p24 as population doubling, using (logN − logN_0_)/log2, where N = confluent cells number and N_0_ = seeded cell number. Live cells were counted by haemocytometer. Viability was assessed by the conventional MTT (3-[4,5-dimethylthiazol-2-yl]-2,5-dephenyl tetrazolium bromide) reduction assay after washing the cells with PBS. Results were expressed as the percentages of reduced MTT, assuming the absorbance of control cells as 100%.

### 2.3. Glutathione Determination

The concentration of glutathione (GSH) was determined in 1 × 10^6^ whole cell lysate after perchloric acid precipitation using the dithionitrobenzoic acid (DTNB) method at 412 nm (molar extinction coefficient 13.6 mM^−1^ cm^−1^), as previously described [14].

### 2.4. Sample Preparation and In-Solution Digestion

For proteomic analysis, 100 µg of protein lysates from untreated and H_2_O_2_-treated ARPE-19 cells were precipitated overnight with ice-cold acetone (1:4 *v*/*v*). The sample preparation for mass spectrometry was carried out using a commercially available in-StageTip (iST) kit including all reagents for lysis, digestion and peptide purification (PreOmics GmbH, Martinsried, Germany). The protein pellet obtained from acetone precipitation was solubilised with the lysis buffer of the iST kit, protein digestion and purification of the peptides were performed according to manufacturer instructions.

### 2.5. LC-MS/MS Analysis and Bionformatic Analysis

Extracted peptides (~1 µg) were separated on a reverse phase PicoFrit column (75 um ID, 8 Um tip, 250 mm bed packed with Reprosil-PUR C18-AQ, 1.9 μm particle size, 120 Å pore size, New Objective, Inc., Woburn, MA, USA, cat. PF7508-250H363), using an EASY-nLC™ 1200 System (Thermo Fisher Scientific, Waltham, MA, USA). Total run time for each sample was 120 min, peptides were separated using a 100 min gradient (4–40% acetonitrile +0.1% formic acid at 300 nL/min). Eluting peptides were measured on-line with a Q Exactive HF benchtop mass spectrometer (Thermo Fisher Scientific) operating in data-dependent acquisition mode (Top20). Peptides were ionized at a potential of +2 KV, intact peptide ions were detected at a resolution of 120,000 (at *m*/*z* 200) and fragment ions at a resolution of 15,000 (at *m*/*z* 200); AGC Target settings for MS were 3E6 charges and for MS/MS 1E5 charges. Peptides were selected for Higher-energy C-trap dissociation (HCD) fragmentation with a quadrupole isolation window of 1.4 Th, peptides were fragmented at a normalized collision energy of 30. The intensity threshold was set at 2E4 and Dynamic exclusion at 30 s. Raw files from MS analysis were processed using the MaxQuant software version 1.6.10.43 22 (Martinsried, Germany) [15]. The spectra were searched against a Uniprot human database (release 2020_2, 20,375 sequences, including isoforms). Precursor and fragment mass tolerance were set to 7 and 20 ppm., respectively, while the minimum length of the peptide was set to 7. False discovery rate (FDR) was set to 1% both at the peptide and protein level. Enzyme for in silico digestion were set to trypsin and lysine C, and up to two missed cleavages were allowed. Cysteine carbamidomethylation (Cys, +57.021464 Da) was set as a fixed modification, whereas *N*-acetylation of proteins (*N*-terminal, +42.010565 Da) and oxidized methionine (Met, +15.994915 Da) were included as variable modifications.

Statistical analysis and plotting were performed using the R language. Protein groups file from MaxQuant was analysed using in-house scripts on label-free quantification intensities (LFQ). Limma R package [16] was used to find differentially expressed proteins (at least ±1.5 fold change and *p*-value < 0.05). To exclude very low abundance proteins, we filtered the dataset excluding all protein groups entries with a sum of peptide-spectral matches (PSMs) across all the samples less than 4. Data were imputed using the QRLIC method included in the imputeLCMD R package [17]. To obtain functional networks of the identified proteins the STRING database was used as previously reported [18,19]. Briefly, the interaction networks were exported as text file and visualized in Cytoscape (v2.8.3) (san Francisco, CA, USA). The combined score from STRING was used to describe the interaction strength. The final network was clustered with the MCL algorithm embedded in the clusterMaker Cytoscape plugin. Functional enrichment was analysed with Bingo Cytoscape plugin and clusterProfiler R package as previously reported [18,20]. The mass spectrometry proteomics data have been deposited to the ProteomeXchange Consortium via the PRIDE [21] partner repository with the dataset identifier PXD022545.

### 2.6. ATP Assay

ATP was evaluated by a Bioluminescent Assay Kit (FL-AA, Sigma-Aldrich, St. Louis, MO, USA). ATP levels were quantified by a calibrated standard curve.

### 2.7. Fluorimetric Determination of Cellular and Mitochondrial ROS

Mitochondrial superoxide ion was determined in 1 × 10^6^ trypsinized cells stained with 5 µM MitoSOX Red (λ_ex_ 510 nm, λ_em_ 600 nm). Fluorescence was detected by a Shimazu RF-500 spectrofluorometer at 37 °C under continuous stirring. Intracellular ROS were quantified by 10 µM 2′,7′-dichlorodihydrofluorescein diacetate (H_2_DCFDA). The fluorescence of 2′,7′-dichlorofluorescein (DCF) was detected by using a Titertek Fluoroscan II (Flow Laboratories, McLean, VA, USA) (λ_ex_ = 485 nm; λ_em_ = 535 nm). Results were expressed as percentage of the control DCF fluorescence normalized to cell viability.

### 2.8. Cytofluorimetric Detection of Mitochondrial Membrane Potential (ΔΨm)

1 × 10^6^ trypsinized cells were loaded with JC-1 (1.5 μM) at 37 °C for 30 min. Evaluation of changes in Δψm was performed by flow cytometry analysis using an EPICS XL-MCL (Beckman Coulter, Brea, CA, USA) as previously described [22]. Green/red fluorescence emission (FL1/FL2) of particles was reported as a dot plot. The uncoupler valinomycin (1 μM) was used to provide depletion of Δψm (positive control).

### 2.9. Oxygen Consumption

Rate of oxygen consumption was determined with an oxytherm Clark-type electrode (Hansatech Instruments, Norfolk, UK). Briefly, after treatment, 5 × 10^6^ trypsinized cells were resuspended in 1 mL of respiration buffer (137 mM NaCl, 5 mM KCl, 0.7 mM NaH_2_PO_4_, 10 mM glucose, and 25 mM Tris-HCl, pH 7.4). Oxygen concentration was determined into a thermostatic chamber at 37 °C under continuous stirring in a buffer containing at first about 200 nmol oxygen/mL at 37 °C.

### 2.10. Western Blot Analyses

Cells were processed with NE-PER^®^ Nuclear and Cytoplasmic Extraction Reagents (Pierce Biotechnology, Rockford, IL, USA) according to manufacturer’s instruction to obtain nuclear extracts; or with RIPA buffer added with protease and phosphatase inhibitors to obtain total cell lysates. Nuclear extracts (30 μg) or total cell lysates (20–40 μg) were separated by 12% or 14% sodium dodecyl sulfate-polyacrylamide gel electrophoresis (SDS-PAGE) and transferred to a nitrocellulose membrane. Non-specific binding sites were blocked in Roti-Block (Roth GmbH, Karlsruhe, Germany) or 5% non-fat milk for 1 h at room temperature. The membranes were blotted overnight at 4 °C with the primary antibodies (Supplementary Table S1). After washing with TBST, blots were incubated for 1 h at room temperature with the appropriate HRP-conjugated secondary antibody and revealed using the enhanced chemi-luminescence (ECL) system (Amersham Pharmacia Biotech, Milan, Italy). Membranes were stripped and re-probed with anti-β-actin, anti-α-tubulin or anti-lamin B as a loading control. Densitometric analyses were performed with ImageJ software (https://imagej.nih.gov/).

### 2.11. Real-Time PCR

Total RNA was extracted using TRIreagent according to the manufacturer’s instructions. cDNA was synthesized from 1 μg total RNA using PrimeScript TM RT Reagent Kit (Takara Bio Europe, Saint-Germain-en-Laye, France). First-strand cDNA synthesis for miRNA was performed using a specific stem loop primer, to selectively transcribe mature miRNA molecules. QPCR was carried out using SYBR Green Jump Start, according to the manufacturer’s instructions, in a 25 μL of reaction volume in presence of 50 ng of cDNA and 400 nM primer. U6 snRNA and GAPDH were used as housekeeping reference for miRNA and mRNA quantification, respectively. In each assay, no-template controls were included, and each sample was run in triplicate. The n-fold differential ratio increase was expressed as 2^−ΔΔCt^. PCR primer sequences, obtained from Bio-Fab research (Rome, Italy) are listed in Supplementary Table S2.

### 2.12. Fluorescence Microscopy Analyses

The immunofluorescence staining of zonula occludens-1 (ZO-1, also called tight junction protein-1) was performed as follows. Cells, grown on polylysine-treated coverslips, were washed in PBS, fixed in ice-cold methanol for 7 min at −20 °C, permeabilized with 1% triton X-100 for 5 min at room temperature. Non-specific binding sites were blocked with blocking buffer (3% BSA in PBS) for 1 h at room temperature. Cells were labelled with anti-ZO-1 primary monoclonal antibody (1A12) (Invitrogen) (1:50) overnight at 4 °C in a humidified atmosphere follower by alexafluor-488 anti-mouse secondary antibody (Invitrogen) for 1 h at room temperature. After washings in PBS added with1% tween-20, nuclei were counterstained with 4,6′-diamidino-2-phenylindole (DAPI).

Morphological changes of plasma membranes were analyzed in live cells, grown on polylysine-treated coverslips, were incubated for 10 min with CellMask red (Thermo Fisher Scientific) in cell culture medium following manufacturer’s instruction. After washings in PBS, nuclei were counterstained with DAPI.

Cells were then rinsed in PBS, mounted and analysed with a Zeiss Axio Observer Z1 equipped with Apotome and digital Camera Axiocam MRm (Zeiss, Oberkochen, Germany).

### 2.13. In Situ Staining for β-Galactosidase Activity

Cells were washed in PBS, fixed with 4% formaldehyde, and incubated overnight at 37 °C in freshly prepared staining buffer [1 mg/mL X-gal (5-bromo-4-chloro-3-indolyl β-D-galactoside), 5 mM K_3_Fe[CN]_6_, 5 mM K_4_Fe [CN]_6_, and 2 mM MgCl_2_, 150 mM NaCl in citrate-buffered saline, pH 6]. At the end of the incubation, cells were washed with H_2_O and examined with an inverted microscope (Leica, Wetzlar, Germany) at 20× magnification.

### 2.14. Statistical Analysis

All results were confirmed in at least three separate experiments and expressed as mean ± S.D. Data were analysed for statistical significance by Student’s *t*-test or two-way ANOVA when appropriate. *p*-values less than 0.05 were considered significant.

## 3. Results

### 3.1. Chronic Exposure to Pro-Oxidant Insult Increases Antioxidant Defences in ARPE-19 Cells

The highly pro-oxidant retinal microenvironment might influence viability and functions of RPE cells [6,7]. Therefore, we investigated the consequences of H_2_O_2_-exposure in ARPE-19 cells. First, we determined the effects of an acute H_2_O_2_ exposure by treating ARPE-19 cells with increasing concentrations (50–800 μM) of H_2_O_2_ for 48 h and determined cell viability (Figure 1B).

We confirmed that ARPE-19 are resistant to acute oxidative stress [23] since doses up to 800 μM only reduced cell viability to 50% of the control.

It is well known that the RPE cell layer is not usually exposed to acute high-dose oxidative stress but, more frequently, to low-level chronical insults [7]. For this reason, we treated ARPE-19 cells with a sublethal dose (10 μM) of H_2_O_2_. Based on morphological observations, we chose to proceed with further experimental procedures after the cells were cultured in the presence of H_2_O_2_ for at least 18 passages (Figure 1A). We followed cell growth during the subsequent period and as shown in Figure 1C, the chronic exposure to low dose H_2_O_2_ did not significantly affect the proliferation rate of ARPE-19 cells. In fact, the growth curve of H_2_O_2_-treated cells is superimposable to that of untreated control cells grown in parallel in the absence of H_2_O_2_. This result suggests that ARPE-19 cells can cope with the chronic sublethal oxidative insult, possibly by increasing cellular antioxidant defenses.

To test this hypothesis, we analysed the nuclear translocation of Nrf2, the master transcription factor devoted to increase the expression of antioxidant defences [2,3]. Surprisingly, we found that a chronic, sublethal oxidative insult decreased Nrf2 nuclear translocation (Figure 1D). We asked whether this result could depend on our experimental setup, since the cells used for the experiments were seeded in H_2_O_2_-free medium and grown for 72 h before harvesting. Therefore, we treated cells with 10μM H_2_O_2_ 6 h before harvesting to simulate an acute treatment. We found that the pro-oxidant insult induced an increase in nuclear translocation of Nrf2 in control cells but not in H_2_O_2_-treated cells (Figure S1) suggesting that a long-term sublethal oxidative insult hampers Nrf2 activation. However, the observed maintenance of cell viability prompted us to analyse the expression of antioxidant defences (Figure 1E). We found that mRNA expression of cystine-glutamate antiporter (xCt), NAD(P)H:quinone oxidoreductase (NQO1), heme oxygenase-1 (HO-1) was increased in H_2_O_2_-treated cells (Figure 1E). We also observed an increase in protein expression of the mitochondrial superoxide dismutase 2 (SOD2) (Figure 1F). Moreover, H_2_O_2_-treated ARPE-19 cells showed a two-fold higher GSH level compared to untreated cells (Figure 1G) thus supporting the protection toward oxidative insults. The observed increase in antioxidant defences correlated with the finding that the viability of H_2_O_2_-treated cells, measured by MTT assay, was similar to that of control cells (Figure 1H). We then evaluated total ROS levels by DCF fluorescence (Figure 1H). We found that the chronic exposure to H_2_O_2_ did not significantly affect intracellular ROS levels. On the other hand, we found a significant decrease in mitochondrial superoxide anion generation (Figure 1J), suggesting that a long-term exposure to sublethal doses of H_2_O_2_ can alter mitochondrial homeostasis presumably by affecting the mitochondrial electron transport chain.

### 3.2. Effects of H_2_O_2_ Chronic Treatment on ARPE-19 Cells Proteome

**Figure 2:**
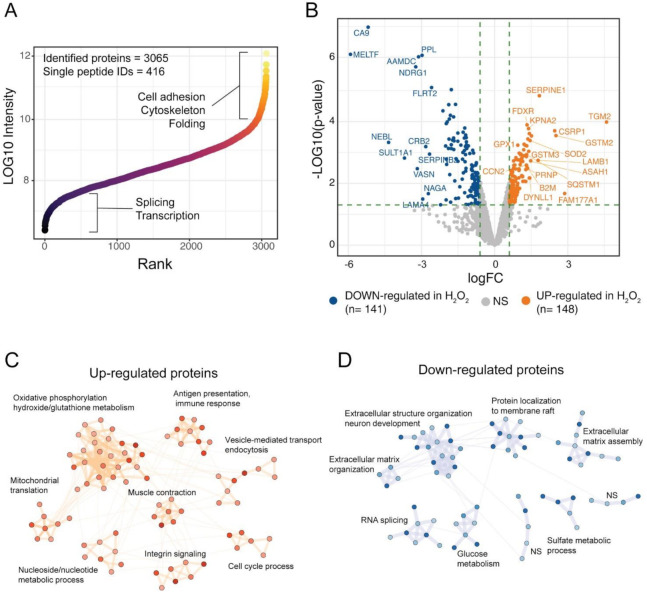
Proteomic analysis of H_2_O_2-_treated ARPE cells. (**A**) the scatter plot reports the identified proteins ranked according to intensity values, ~3000 protein groups were identified in ARPE-19 cells, across six orders of magnitude; (**B**) Volcano plot of the differentially expressed proteins upon H_2_O_2_ treatment. A total of 148 proteins were up-regulated by H_2_O_2_ treatment, while 141 showed down-regulation as compared to control samples; (**C**,**D**) Network analysis of DE proteins after H_2_O_2_ treatment. Networks were retrieved using the STRING database and visualised using the Cytoscape software. Functional enrichment was used to understand which biological processes were enriched in each cluster.

To get further insights on the response of ARPE-19 cells to the chronical oxidative insult induced by H_2_O_2_ we performed a global proteomic analysis of our RPE cell model. The proteome changes induced by chronic treatment of ARPE-19 cells with H_2_O_2_ were studied by high-resolution LC-MS/MS and differential protein expression analysis using label-free quantification (LFQ). By using mass spectrometry analyses, we identified 3065 protein groups across the three technical replicates of the two experimental groups (Ctr and H_2_O_2_), of these, 416 were single-peptide identifications while the remaining protein groups were identified with more than 1 peptide (Figure 2A). The identified proteome spanned across six orders of magnitude, showing a good depth of the single-shot proteomic analysis (Figure 2A). High-abundance proteins were linked to cell adhesion, cytoskeleton and folding, while low-abundance proteins were mostly involved in splicing and transcription (Figure 2A). Protein abundances were investigated using LFQ intensities from the MaxQuant computational platform, while differential expression (DE) analysis was performed using limma algorithms. DE analysis showed 148 up-regulated proteins upon treatment with H_2_O_2,_ while 141 protein groups were down-regulated, as compared to Ctr samples (Figure 2B). Among the proteins with highest up-regulation were the protein-glutamine gamma-glutamyltransferase 2 (TGM2), the protein FAM177A1 and the Glutathione S-transferase Mu 2 (GSTM2). Among the down-regulated proteins melanotransferrin (MELTF) and carbonic anhydrase 9 (CA9) showed the largest decrease in H_2_O_2_ treated cells (Supplementary Table S3). To understand the biological processes in which the DE proteins might be involved, we performed a network analysis using the STRING database and investigated the obtained network using cluster analysis and functional enrichment. Results are reported in Figure 2C,D; the complete networks are reported in Supplementary Figures S2 and S3. The over-expressed proteins after H_2_O_2_ treatment showed 8 clusters with significant association with biological processes (Figure 2C). The largest cluster was linked to oxidative phosphorylation and oxidative stress, the majority of proteins in this cluster belonged to the mitochondrial electron transport chain. Other two clusters were involved with mitochondria organization and energy metabolism, one was composed of proteins linked to mitochondrial translation, the other one to ATP metabolic processes. Other clusters of the up-regulated proteins were linked to immune response and antigen presentation, endocytosis, and integrin signaling (Figure 2C). The network of down-regulated proteins in H_2_O_2_-treated cells was composed mainly of proteins involved in extracellular matrix organization. Several collagen proteins (COL12A1, COL11A1, COL18A1, cluster 9, Figure S2) showed down-regulation in the H_2_O_2_-treated sample, together with laminins (LAMC1, LAMA4, LAMB2), and other proteins involved in matrix and cytoskeleton remodelling, such as the microtubule-associated protein 2 (MAP2) the glypican (GPLC1) and agrin (AGRN, cluster 1, Figure S2). These data suggest that a long-term exposure to oxidative insults impairs the ability of RPE cells to correctly synthesise Bruck’s membrane proteins thus potentially compromising the transport of waste to the choriocapillaris [4]. Down-regulated proteins were also involved in membrane raft composition and function, glucose metabolism, RNA splicing and sulfate metabolism in cells (Figure 2D and Figure S3).

### 3.3. Chronic Sublethal Oxidative Insult Induces Mitochondrial Alterations Linked to Senescence in ARPE-19 Cells

**Figure 3:**
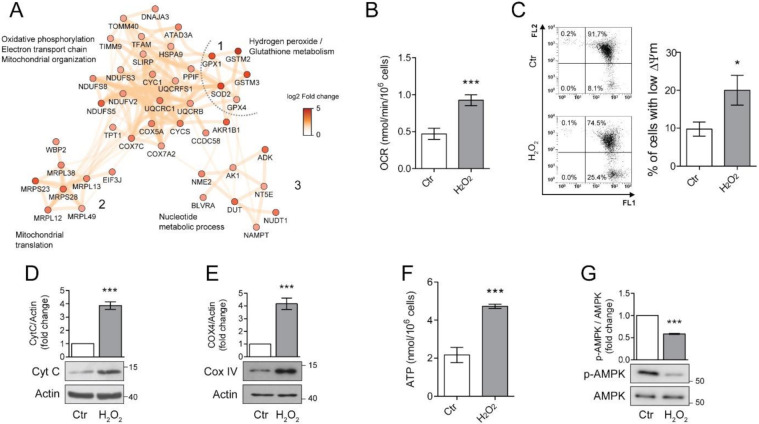
Chronic sublethal oxidative insult induces mitochondrial alterations in ARPE-19 cells. (**A**) Network of up-regulated protein clusters involved in mitochondrial and energy-related processes. Cluster 1 was linked to oxidative phosphorylation, electron transport and mitochondrial organization, cluster 2 included several proteins involved in mitochondrial translation, while cluster 3 was composed of proteins involved in nucleotide metabolism. Color is related to the log2-fold change difference in expression between H_2_O_2_ and Ctr. Ctr and H_2_O_2_ treated cells were seeded in H_2_O_2_-free medium, grown for 72 h and used for the experiments; (**B**) Oxygen concentration was recorder in 1 mL of respiration buffer containing 200 nmol oxygen/mL at 37 °C. Data are expressed as nmol/min/10^6^ cells and are the mean ± SD of four independent experiments; (**C**) Cells loaded with 1.5 µM JC-1 for 30 min and analysed by flow cytometry. Representative dots (upper panel) represent a single cell analysed for its green (FL1) and orange (FL2) associated fluorescence. Number in each quarter indicate the cell population percent. In the histogram, data represent the mean ± SD of three independent experiments; (**D**,**E**) Western blotting analysis of Cytochrome C (Cyt C) and Cytochrome oxidase IV (COXIV), respectively. β-actin was used as loading control. Bar graphs represent the densitometric analysis reported as the ratio between protein and β-actin band intensity. Untreated cells were used as control and assumed as 1. One representative blot is shown and densitometric analyses are expressed as the mean ± SD of three independent experiments; (**F**) Total cellular ATP levels measured by ATP luminescence-based assay and reported as nmol/10^6^ cells; (**G**) Western Blotting of phosphorylated and total AMPK. Bar graphs represent the densitometric analysis reported as the ratio between phosphorylated and unphosphorylated AMPK band intensity. Untreated cells were used as control and assumed as 1. One representative blot is shown and densitometric analyses are expressed as the mean ± SD of three independent experiments (* *p* < 0.05, *** *p* < 0.001).

Proteomic analysis revealed a significant alteration in mitochondrial homeostasis which is one of the key features of the senescence associated phenotype (SAP) [11]. To better understand the involvement of mitochondria in long-term H_2_O_2_ response, we focused our attention of three clusters of proteins associated with mitochondrial organization and energy relate processes (Figure 3A). We found that chronic exposure to H_2_O_2_ induced the up-regulation of 12 subunits of the mitochondrial electron transport/oxidative phosphorylation (OXPHOS) pathway, with 4 subunits from the NADH dehydrogenase (complex I), 3 subunits of the cytochrome C reductase (complex III) and 4 subunits of the cytochrome C oxidase (complex IV). Additionally, also cytochrome C (CYCS), was overexpressed in H_2_O_2_ treated ARPE-19 cells (log2 fold change = 0.99, Figure 3A, cluster 1).

The same cluster also contains a group of proteins involved in mitochondrial oxidative stress, these included superoxide dismutase 2 (SOD2), glutathione peroxidase 1 (GPX1), glutathione peroxidase 4 (GPX4). The over-expression of these proteins confirms the induction of a protective phenotype against oxidative stress after H_2_O_2_ treatment. The mitochondrial alterations were not limited to the oxidative phosphorylation process but also included the overexpression of a cluster of 6 mitochondrial ribosomal proteins (MRLPs), involved in the translation of mitochondrial proteins (cluster 2, Figure 3A). Additionally, proteins linked to mitochondrial organization and biogenesis were also over-expressed, such as the mitochondrial transcription factor A (TFAM), stomatin-like protein 2 (STOML2) and ATPase family AAA domain-containing protein 3A (ATAD3A, Figure 3A cluster 1) [24,25,26]. Finally, an up-regulation of a cluster of proteins linked to nucleoside metabolism was also noticed (Figure 3A, cluster 3). This cluster included two important enzymes for ATP metabolism, adenosine kinase (ADK) and adenylate kinase 1 (ADK1). Taken together, these results show that H_2_O_2_ chronic treatment induced a dramatic reorganization of the mitochondrial processes, possibly including ATP generation, mitochondria biogenesis and response to oxidative stress.

Since increased mitochondrial biogenesis and respiration are considered important hallmarks of senescence [11], we sought to validate these findings in our model of chronic sublethal oxidative insult of the RPE. First, we performed an immunoblot analysis of Cyt C (Figure 3B) and Cytochrome oxidase 4 (COX IV) (Figure 3C) to validate the increased expression of protein belonging to the electron transport chain found by proteomics. Levels of Cyt C and COX IV were significantly increased in H_2_O_2_-treated ARPE-19 cells, confirming the up-regulation of the OXPHOS pathway. Next, we measured oxygen consumption rate (OCR), mitochondrial membrane potential (∆ψm) and ATP production in our experimental conditions. We found that the rate of oxygen consumption (Figure 3D) was twice as higher in H_2_O_2_-treated cells compared to the untreated counterpart even if a small, although significant increase in the number of cells with a reduced ∆ψm was observed (Figure 3E). These results are in line with the observed doubling in ATP production (Figure 3F) and the decrease in expression of the phosphorylated/active form of AMPK (Figure 3G) which indicates a high ATP/AMP ratio [27].

**Figure 4:**
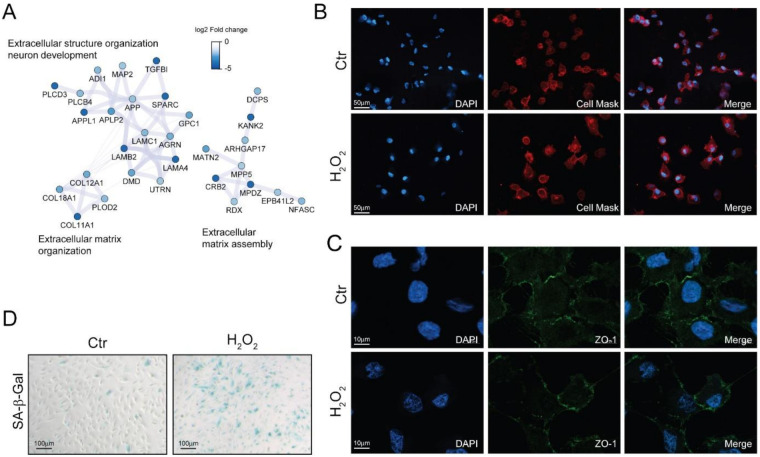
Chronic sublethal oxidative insult induces senescence in ARPE-19 cells. (**A**) Selected networks of proteins showing down-regulation in H_2_O_2_-treated cells and involved in extracellular matrix organization and assembly. Node color is related to the log2 fold change difference in expression between H_2_O_2_ and Ctr. Ctr and H_2_O_2_ treated cells were seeded on coverslips H_2_O_2_-free medium and grown in for 72 h before processing; (**B**) Morphological changes of plasma membranes in live cells assessed by CellMask Red staining; magnification 20×; (**C**) Expression and distribution of ZO-1 determined by immunofluorescence analysis. Magnification 100×; (**D**) SA-β-Gal activity assay. Senescent cells are stained in blue, magnification 10×. In (**B**–**D**) the images are representative of one out of three separate experiments.

Morphological alterations are another fundamental feature of senescent cells [11]. The down-regulation of cytoskeletal and extracellular matrix proteins observed with proteomics (Figure 4A) may induce alteration in the cell shape. Indeed, investigating cell morphology by fluorescent microscopy, we found that H_2_O_2_-treated cells were enlarged and showed an elongated phenotype (Figure 4B), however no significant alterations in the distribution of the tight-junction protein zonula occludens-1 (ZO-1) could be appreciated (Figure 4C).

Senescent cells exhibited increased activity of the acidic senescence-associated β-galactosidase (SA-β-Gal) [11]. We found an increase in SA-β-Gal positive cells upon long-term sublethal oxidative insult exposure (Figure 4D) thus supporting the induction of a senescent associated phenotype. Interestingly, we also found that SA-β-Gal positivity increased together with the time of exposure to H_2_O_2_ and that an acute treatment with *N*-acetyl cysteine partially reverted the increase in SA-β-Gal positivity in a dose-dependent manner (Figure S4).

### 3.4. Senescent ARPE-19 Cells Show an Inflammatory Phenotype

**Figure 5:**
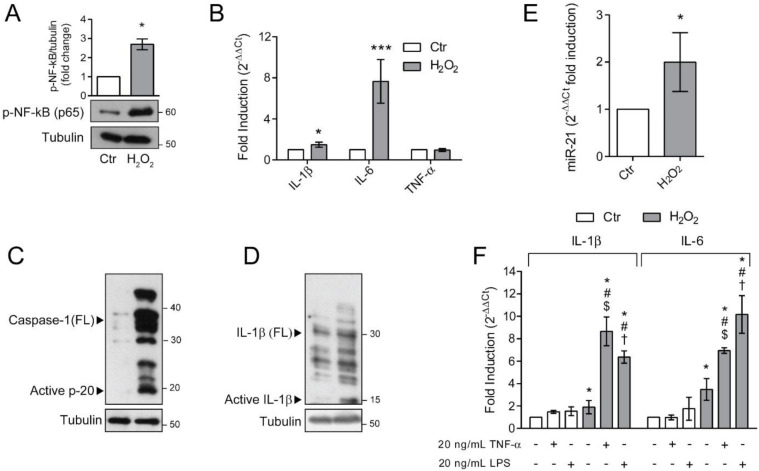
Senescent ARPE-19 cells show an inflammatory phenotype. Ctr and H_2_O_2_ treated cells were seeded in H_2_O_2_-free medium and grown for 72 h and used for experiments (**A**) Western Blotting of phosphorylated NF-κB p65. α-tubulin was used as loading control. Bar graphs represent the densitometric analysis reported as the ratio between protein and α-tubulin band intensity. Untreated cells were used as control and assumed as 1. One representative blot is shown and densitometric analyses are expressed as the mean ± SD of three independent experiments; (**B**) gene expression of IL-1β, IL-6 and TNF-α assessed by qPCR. Gene expression values were normalized to GAPDH and presented as 2^−ΔΔCt^. Relative mRNA gene abundance in untreated cells was assumed as 1 (control). Data represent the mean ± SD from *n* = 5 independent experiments performed in triplicate. (* *p* < 0.05, *** *p* < 0.001); (**C**,**D**) Western Blot analysis of Caspase-1 and IL-1β full length (FL) and cleaved active fragments. α-tubulin was used as loading control. Bar graphs represent the densitometric analysis reported as the ratio between protein and α-tubulin band intensity. Untreated cells were used as control and assumed as 1. One representative blot is shown and densitometric analyses are expressed as the mean ± SD of three independent experiments; (**E**) Expression of miR-21 by qPCR. Gene expression values were normalized to U6snRNA and presented as 2^−ΔΔCt^. Relative mRNA gene abundance in untreated cells was assumed as 1 (control). Data represent the mean ± SD of three independent experiments; (**F**) Ctr and H_2_O_2_-treated cells were exposed to 10 μg/mL LPS or 20 nM TNFα for 6 h before harvesting. Gene expression of IL-1β and IL-6 was assessed by qPCR. Gene expression values were normalized to GAPDH and presented as 2^−ΔΔCt^. Relative mRNA gene abundance in untreated cells was set to 1 (control). Data represent the mean ± SD from n = 3 independent experiments performed in triplicate. (* at least *p* < 0.05 vs. Ctr non-treated (--); # *p* < 0.05 vs. H_2_O_2_ non-treated (--); $ *p* < 0.05 vs. Ctr TNFα + LPS-; † *p* < 0.05 vs. Ctr TNFα − LPS +).

Senescent cells are characterized by a distinctive senescence-messaging secretome or SASP which includes a plethora of pro-inflammatory mediators [11]. To address whether ARPE-19 cells, rendered senescent by long-term exposure to H_2_O_2_, were characterized by a pro-inflammatory phenotype, we first determined the activation of the master regulator of inflammation, NF-κB (Figure 5A). We found a dramatic increase in the phosphorylated form of NF-κB p65 subunit in H_2_O_2_-treated ARPE-19 cells. The activation of NF-κB correlates with the observed increase in the expression of pro-inflammatory genes IL-6 and IL-1β (Figure 5B). In the same experimental condition, TNF-α expression was not modified (Figure 5B). The increase in NF-κB activation and IL-1β expression prompted us to evaluate inflammasome activation. To this end, we determined the expression of full-length (FL) and cleaved Caspase-1 (Figure 5C) and IL-1β (Figure 5D). Chronic sublethal H_2_O_2_ treatment induced a remarkable increase in the expression of FL Caspase-1 and cleaved p20 active form, which correlated with the observed increase in the mature p10 form of IL-1β. These results support the acquisition of a pro-inflammatory phenotype by senescent ARPE-19 cells. It is known that NF-κB, beside regulating the expression of pro-inflammatory cytokines, also positively regulates miR-21 expression under oxidative stress conditions [28] and that a dysregulated miR-21 expression is associated with numerous retinal diseases [29,30,31]. Therefore, we analysed miR-21 expression in our experimental conditions (Figure 5E) and found that chronic H_2_O_2_ exposure doubled miR-21 expression in ARPE-19 cells.

The increased inflammatory phenotype observed in H_2_O_2_-treated ARPE-19 cells, fostered us to analyse the effects of additional exogenous inflammatory stimuli, both pathogen-associated and sterile. To this aim, we treated either control or senescent ARPE-19 cells with 10 μg/mL LPS or 20 ng/mL TNFα and measured the expression of pro-inflammatory cytokines IL-6 and IL-1β (Figure 5F). We found that the exposure of H_2_O_2_-treated cells to the inflammatory stimuli induced a prominent increase in IL-1β and IL-6 expression compared to the untreated counterpart. These data indicate that the cellular alterations induced by chronic sublethal oxidative insult render ARPE-19 cells more responsive to pro-inflammatory insults.

## 4. Discussion

In this work, we demonstrated, for the first time, that a long-term sublethal oxidative insult induces a senescence-associated phenotype in ARPE-19 cells. This is characterised by alterations in mitochondrial metabolism, extracellular matrix organization, inflammasome activation and exaggerated inflammatory response.

Both oxidative stress and inflammation have been associated with the pathogenesis of age-related macular degeneration (AMD) [2]. Antioxidant supplementation is the only available intervention for AMD, [5] strongly implicating oxidative stress in the pathogenesis of the disease. Moreover, oxidative stress has also been indicated as of one of the main culprits in other retinopathies including retinite pigmentosa, for which treatment based on the antioxidant *N*-acetylcysteine has shown efficacy in improving cone function in a randomized, placebo-controlled, phase I trial [32].

It must be stressed that, when testing the involvement of oxidative stress in retinal diseases, the majority of in vitro studies employed lethal pro-oxidant insults and in the majority of cases the treatment lasted for no more than one week (reviewed in [33]). As an example, H-RPE cells (Human Retinal Pigment Epithelial Cells) exposed to the oxidant agent *N*-retinylidene-*N*-retinylethanolamine (A2E) for 3 and 6 h, display a dramatic reduction in cell viability [34]. Here, instead, we analyzed the effects of a sublethal dose of H_2_O_2_ protracted for 18–24 cell passages on a cell model of the RPE. This model more closely mimics the chronic oxidative insults typical of the human eye during aging. Indeed, RPE cells coexist, throughout life, with moderate sublethal levels of pro-oxidant molecules that are generated by both extracellular and intracellular processes [6,8].

Additionally, also genetic traits linked to oxidative stress have been associated to AMD. For example, polymorphisms of NADH dehydrogenase subunits and SOD2 have been linked to the pathogenesis of AMD [35,36]. In our experimental setting, both NADH dehydrogenase subunits and SOD2 protein levels were increased strengthening the reliability of our experimental model.

A life-long exposure to a pro-oxidant milieu might also impair RPE cell functions including the ability to correctly synthesise Bruck’s membrane components, which, in turn, can be involved in drusen deposition [4]. Our results showing the downregulation of several collagen and laminin proteins strongly indicate that our experimental model is able to mimic different features of AMD [4].

We have shown that the chronic sublethal exposure to a pro-oxidant stimulus induced a senescence-associated phenotype in ARPE-19 cells. After passaging the cells for 18–24 times in the presence of H_2_O_2_, we obtained a senescent cell line, as shown by the observed increase in SA-β-Gal activity. These cells, however, were characterized by a replicative senescent phenotype as evidenced by the maintenance of a population doubling rate comparable to that of untreated cells grown under the same experimental conditions but in the absence of H_2_O_2_.

The role of cell senescence in ocular diseases has recently received much attention [11], even though the presence of SA-β-Gal positive RPE cells in human and monkey retina has been reported more than 20 years ago [37]. More recently, an increase of canonical senescence markers in the RPE isolated from aged human donors was demonstrated [38], further supporting the involvement of senescence in age-related retinal diseases. Besides the increase in SA-β-Gal activity, the senescent phenotype is characterized by precise features. Among those, significant morphological alterations including enlargement and flattening are often present in senescent cells [11]; these changes were also present in our cell model system. Another hallmark of senescence is the alteration of mitochondrial homeostasis, characterised by increased mitochondrial biogenesis and respiration [9]. Proteomic analyses of ARPE-19 cells chronically exposed to H_2_O_2_ at low doses, evidenced an increase in the expression of proteins linked to mitochondrial homeostasis. For example, we found an increased expression of a protein cluster including several mitochondrial ribosomal proteins, such as the 39S ribosomal protein L13 and 28S ribosomal protein S23. The upregulation of these proteins is indicative of a higher mitochondrial translation rate and suggests an increase in mitochondrial biogenesis. This observation is also strengthened by the increased expression of COX IV, Cyt C and of the transcription factor TFAM, which has a critical role in the initiation of mitochondrial transcription [26]. Protein involved in mitochondrial metabolism were also upregulated by chronic H_2_O_2_ treatment. The substantial increase of several components of the oxidative phosphorylation (OXPHOS)/electron transport chain (ETC) found by proteomics is indicative of an increased availability of key proteins involved in ATP synthesis. In fact, H_2_O_2_ exposed cells showed a twice as high ATP level as untreated cells. The availability of sufficient ATP amounts at the cellular level is confirmed by the decreased activation of AMPK, which is responsible for the concerted regulation of catabolism and anabolism depending on cellular energy levels [27]. The reliance on mitochondrial ETC for ATP production is supported by the finding that OCR was increased in H_2_O_2_-treated cells. However, a significant reduction in mitochondrial membrane potential was observed upon long-term H_2_O_2_ treatment, which seems to indicate the existence of a dysfunctional mitochondrial sub-population. Dysfunctional mitochondria are characterized by leak of electrons from ETC, which causes an increase in mitochondrial ROS production [39]. Paradoxically, in our experimental model system, we found a decrease in MitoSox fluorescence indicative of a lower mitochondrial superoxide anion levels. This finding can be explained by the observed increase in the mitochondrial SOD2, which is devoted to the dismutation of superoxide anion, the main product of electron leakage, into the less harmful hydrogen peroxide. Nonetheless, the intracellular ROS levels, detected by DCF fluorescence, are not modified by H_2_O_2_ treatment. This result is in line with the observed increase in the expression of genes involved in the antioxidant response. Proteomic analysis also revealed an increase in the abundance of antioxidant-related proteins. In particular, the increase of Biliverdin reductase A (BLVRA) protein expression correlates with the increase in HO-1 gene expression. HO-1 catalyses the first, rate-limiting step in the degradation of pro-oxidant heme molecule yielding equimolar quantities of carbon monoxide (CO), iron ions (Fe^2+^) and biliverdin. The latter is in turn reduced to non-toxic bilirubin by biliverdin reductase [40].

Proteomic and transcriptional analyses also revealed an increased expression of proteins involved in glutathione metabolism like GPX1, GPX4 and xCt; this increase strongly correlates with the increase in GSH levels measured in H_2_O_2_-treated cells. We can hypothesize that the unchanged cell viability demonstrated in our experimental model rely on the observed increase in antioxidant defences, potentially due to the activation of the transcription factor Nrf2. Indeed, Nrf2 senses intracellular oxidative stress and by binding to the EpRE/ARE (antioxidant/electrophile response element), induces the expression of phase II/antioxidant genes including NQO-1, HO-1 and xCt [41]. Surprisingly, we found that Nrf2 nuclear translocation was lower in H_2_O_2_-treated cells compared to the untreated counterpart and that not even a short-term 10 μM H_2_O_2_ acute exposure was capable of restoring Nrf2 response to the level observed in control cells. Although unexpected, this result is in line with the findings that aging can impair Nrf2 signalling in RPE after an acute oxidative insult [42]. It has also been shown that Nrf2-deficient mice develop an ocular disease that resemble AMD [43], indicating that Nrf2 dysfunction is a pivotal event in age-related retinopathies. However, increase in antioxidant defences can be also sustained by other transcription factors, including nuclear factor-κB (NF-κB). NF-κB is a redox sensing inducible transcription factor mainly devoted to immune and inflammatory response. It has been demonstrated that NF-κB can also control the transcription of genes devoted to the antioxidant response, including SOD2, glutathione S-transferase, NQO-1, GPX1 and HO-1 [44]. The same antioxidant defences were up-regulated in our cell model, suggesting an involvement of NF-κB in the observed phenotype. In fact, we found that H_2_O_2_-treated cells possessed a three times higher phosphorylated/active NF-κB compared to control cells explaining the increase in antioxidant defences in the absence of increased Nrf2. The increase in NF-κB activation might also be the responsible for the lack of Nrf2 activation via the known molecular cross-talk between the two transcription factors [45].

It has been shown that upon oxidative injury, NF-κB can also upregulate the expression of miR-21, which has been involved in diabetic retinopathy and vitroretinal disease [30,31]. Moreover, it has been demonstrated that miR-21, which shows a dramatic age-dependent increase, is loaded on extracellular vesicles and transferred from RPE to retinal microglia where it influences gene expression [46]. Our findings suggest that H_2_O_2_-treated cells produce and potentially secrete bioactive molecules capable of altering the behaviour of neighbouring retinal cells.

The microenvironmental cross-talk among different cell layers of the retina can be further influenced by the acquisition of SASP [9]. A main feature of SASP is the presence of cytokines and chemokines whose expression is regulated by NF-κB [47]. In our experimental model, the long-term exposure to H_2_O_2_ increased the expression of the inflammatory cytokines IL-6 and IL-1β thus suggesting the acquisition of SASP, which could also affect other retinal cells during pathogenesis and progression of AMD [9,48]. Moreover, the increase in pro-inflammatory mediators upon senescence induction may mirror inflammaging, a state of low-grade chronic inflammation induced by age-related mitochondrial dysfunction that is deeply connected to age-relate pathologies including AMD [49,50].

In the presence of these inflammatory conditions, the activation of inflammasome can be envisaged. Indeed, we found that H_2_O_2_-treated cells displayed dramatically increased levels of Caspase-1 both in the full-length form and in the cleaved/active form, which, in turn, correlates with the observed increase in the cleaved/mature form of IL-1β. We showed that, upon long-term sublethal oxidative insult, inflammasome activation does not require additional inflammatory stimuli. It is known that canonical inflammasome activation is a two-step mechanism: a priming step deputed to increase NF-κB activation and an activation step to permit inflammasome platform assembly [51]. In our experimental conditions, the overactivation of NF-κB makes inflammasome activation independent from an external priming stimulus. The activation step, required for inflammasome assembly and Caspase-1 cleavage/activation, can be dependent on a broad range of stimuli including ROS generation, K^+^ efflux or lysosomal destabilization [51]. In our cell model, the increase in SA-β-Gal activity, indicative of lysosomal leakage, may explain the observed increase in active Caspase-1 and the consequent IL-1β maturation in the absence of an activating stimulus. Several experimental in vitro evidences suggest a pivotal role for inflammasome activation in AMD [12,13,52,53]. Moreover, the inflammatory state associated with cell senescence has been shown to prime inflammasome activation in murine lungs [54] and fibroblasts [55].

We also found that a second inflammatory hint, either pathogen-associated or sterile, could further increase cytokine production in senescent ARPE-19 cells. This result correlates with the age-related aberrancies in inflammatory responses observed in mouse models of aging [54] and with the altered expression levels of inflammatory factors detected in the eye of AMD patients [56]. These data indicate that a long-term exposure to sub-toxic oxidative insult, a condition mimicking retinal environment, modify the ability of ARPE-19 cells to endure a pro-inflammatory stimulus. Nevertheless, the use of other RPE cell models will increase the translational relevance of our findings.

## 5. Conclusions

In conclusion, our results infer long-term sublethal oxidative insult as a key factor in the pathogenesis of age-related retinal degeneration such as AMD. During the aging process, the retina endures low-grade chronic oxidative insult, which persists for decades and usually gets worse with advancing age. As a result, microglia and complement system, which represent the retinal innate immune system, experience constant low levels activation [57]. Our results point to the involvement of RPE cells as contributors to the inflammatory milieu of the human aging retina. While a moderate inflammatory response can preserve eye homeostasis during healthy aging, the dysregulated inflammatory response might contribute to the macular damage in AMD affected subjects [9]. The dysregulation of the inflammatory response in the aging retina may depend on several factors including genetic predisposition and environmental agents [2,3]. We showed that inflammation might be among these factors. In fact, the observation that additional inflammatory stimuli induce a further increase in pro-inflammatory cytokines in aged cells may clarify the link between chronic oxidative insult and detrimental chronic inflammation, which are key contributors to AMD pathogenesis and progression.

## Data Availability

The mass spectrometry proteomics data have been deposited to the ProteomeXchange Consortium via the PRIDE [21] partner repository with the dataset identifier PXD022545. The other data are contained within the article and supplementary material.

## Supplementary Materials

The following are available online at https://www.mdpi.com/2076-3921/10/1/25/s1, Table S1: List of the antibodies used in this work; Table S2: List of the primers used in this work; Table S3: Differential protein expression between H2O2-treated and control (Ctr) ARPE-19 cells; Figure S1: Assessment of Nrf2 nuclear translocation by western blotting. Figure S2: Network analysis of up-regulated proteins in H2O2-treated ARPE-19 cells; Figure S3: Network analysis of down-regulated proteins in H2O2-treated ARPE-19 cells; Figure S4: Chronic sub-lethal oxidative insult induces senescence in ARPE-19 cells.
