# Supplementary material for: Crosstalk between Long-Term Sublethal Oxidative Stress and Detrimental Inflammation as Potential Drivers for Age-Related Retinal Degeneration
Source: Antioxidants (Basel). 2020 Dec 29;10(1):25. doi: 10.3390/antiox10010025 (PMC7823845; doi:10.3390/antiox10010025)
Supplement: Supplementary file 1 [file antioxidants-10-00025-s001.pdf]

Supplementary material

Supplementary table S1 List of the antibodies used in this work. The antibody, the host species, the distributor, the product number and the dilution are reported in the table.

Antibody	Host Species	Distributor, Cat. Num.	Diluition WB
Anti-Actin (AC-40) (Actin)	mouse, monoclonal	Sigma-Aldrich, Milan, Italy #A4700	1:1000
Anti-AMPKα (Thr172) (D4D6D) (AMPKα)	rabbit, polyclonal	Cell Signaling Technology, Danvers, USA # 2532	1:1000
Phospho-AMPKα (Thr172) (D4D6D) (p-AMPKα)	rabbit, monoclonal	Cell Signaling Technology, Danvers, USA #50081	1:1000
Anti-Akt (Akt)	rabbit, polyclonal	Cell Signaling Technology, Danvers, USA #9272	1:1000
Anti phospho-Akt (Ser-473) (p-AKT)	rabbit, polyclonal	Cell Signaling Technology, Danvers, USA #9271	1:1000
anti-Caspase-1 (caspase-1)	rabbit, polyclonal	Cell Signaling Technology, Danvers, USA #2225	1:1000
Anti-Cytochrome c (A-8) (cyt c)	mouse, monoclonal	Santa Cruz Biotechnology, Dallas, TX, USA #sc-13156	1:500
Anti-Cytochrome c oxidase (F-8) (COX IV)	mouse, monoclonal	Santa Cruz Biotechnology, Dallas, TX, USA #sc-376731	1:700
Anti-IL-1β (3A6) (IL-1β)	mouse, monoclonal	Cell Signaling Technology, Danvers, USA #12242	1:1000
Anti-Manganese superoxide dismutase (SOD2)	rabbit, monoclonal	Cell Signaling Technology, Danvers, USA #13141	1:1000
anti-Phospho-NF-kB p65 (Ser536) (93H1) (NF-kB)	rabbit, monoclonal	Cell Signaling Technology, Danvers, USA #3033	1:1000
Anti-β-Tubulin (Tubulin)	mouse, monoclonal	Proteintech, Rosemont, USA #66240-1-Ig	1:5000

Supplementary table S2. List of the primers used in this work. Forward and reverse primers for the genes tested in this work are reported in the supplementary table. Additionally, the miR-21 stem loop used is also reported.

Gene name	Forward primer	Reverse primer
**XCt**	CAACTAGAAGCGTGACAGGT	GATGCATGTGCTTTTGTATG
**NQO-1**	GGGTATCTTTCCAGGCTTCC	TTTCTACATCTTTCCCTAAGTGGC
**HO-1**	TGTGGCAGCTGTCTCAAACCTCCA	TTGAGGCTGAGCCAGGAACAGAGT
**GAPDH**	TGGTATCGTGGAAGGACTCATGAC	ATGCCAGTGAGCTTCCCGTTCAGC
**miR-21**	ACACTCCAGCTGGGTAGCTTATCAGACTGA	TGGTGTCGTGGAGTCG
**miR21 stem-loop**	CTCAACTGGTGTCGTGGAGTCGGCAATTCAGTTGAGTCAACATC	

Supplementary table S3 . Differential protein expression between H_2O_2 -treated and control (Ctr) ARPE-19 cells.
The protein groups identified using MaxQuant computational platform were analysed using limma statistics to calculate significance values and fold change of different comparisons. log2FC: log2 fold change H2O2 vs Ctr, AveExpr: average expression across all the samples according to normalised log2 iBAQ intensities, t: moderated t statistic of the limma analysis, P Value: p-value of limma statistics, adj P Value: adjusted p-value of limma statistics according to Benjamini and Hochberg FDR.

Protein name	Gene name	uniprotID	Sequence coverage (%)	Mol_weight kDa	Nr. Peptides	log2FC	AveExpr	t	P.Value	adj.P.Val
Protein-glutamine gamma-glutamyltransferase 2	TGM2	P21980	14.8	77.3	7	4.6	25.5	8.0	1.0E-04	1.4E-02
Protein FAM177A1	FAM177A1	Q8N128	15.5	23.8	3	2.9	23.1	3.0	2.1E-02	1.8E-01
Isoform 2 of Glutathione S-transferase Mu 2	GSTM2	P28161-2	31.4	22.6	4	2.5	25.8	6.8	2.9E-04	2.3E-02
Cysteine and glycine-rich protein 1	CSRP1	P21291	20.7	20.6	3	2.5	25.1	7.2	2.0E-04	2.0E-02
Plasminogen activator inhibitor 1	SERPINE1	P05121	67.4	45.1	20	1.8	30.9	10.9	1.5E-05	3.9E-03
Laminin subunit beta-1	LAMB1	P07942	5.2	198.0	5	1.8	25.7	4.9	1.8E-03	4.7E-02
Isoform 2 of MAP7 domain-containing protein 1	MAP7D1	Q3KQU3-2	10.8	88.7	7	1.5	26.5	6.8	2.9E-04	2.3E-02
Isoform 2 of Acid ceramidase	ASAH1	Q13510-2	14.8	46.5	5	1.5	26.1	4.9	1.9E-03	4.8E-02
Superoxide dismutase [Mn], mitochondrial	SOD2	P04179	53.6	24.8	8	1.5	30.7	6.4	4.1E-04	2.5E-02
Dynein light chain 1, cytoplasmic	DYNLL1	P63167	20.2	10.4	2	1.4	27.7	3.8	6.7E-03	1.0E-01
Isoform 4 of Coiled-coil domain-containing protein 9B	CCDC9B	Q6ZUT6-4	24.7	49.7	9	1.4	27.0	7.0	2.5E-04	2.2E-02
GSK3B-interacting protein	GSKIP	Q9P0R6	38.8	15.6	2	1.4	25.8	3.8	6.7E-03	1.0E-01
Glutathione S-transferase Mu 3	GSTM3	P21266	19.6	26.6	3	1.4	25.6	5.6	9.2E-04	3.4E-02
Importin subunit alpha-1	KPNA2	P52292	35.3	57.9	9	1.4	27.1	7.4	1.7E-04	1.8E-02
OCIA domain-containing protein 2	OCIAD2	Q56VL3	14.3	17.0	2	1.4	26.5	6.5	3.7E-04	2.4E-02
Major prion protein	PRNP	P04156	7.9	27.7	2	1.3	26.3	4.4	3.3E-03	6.7E-02
Isoform 4 of NADPH:adrenodoxin oxidoreductase, mitochondrial	FDXR	P22570-4	16.9	48.1	7	1.3	26.8	7.8	1.3E-04	1.5E-02
Glutathione peroxidase 1	GPX1	P07203	29.6	22.1	5	1.3	26.6	6.0	6.1E-04	2.8E-02
Beta-2-microglobulin	B2M	P61769	31.1	13.7	3	1.3	28.9	4.6	2.8E-03	6.0E-02
Isoform 2 of Sequestosome-1	SQSTM1	Q13501-2	31.7	38.6	7	1.3	27.2	5.3	1.2E-03	3.7E-02
Phostensin	PPP1R18	Q6NYC8	17.8	67.9	7	1.3	26.8	5.2	1.4E-03	4.2E-02
CCN family member 2	CCN2	P29279	6.3	38.1	2	1.3	24.8	4.6	2.6E-03	5.8E-02
ATP synthase-coupling factor 6, mitochondrial	ATP5PF	P18859	35.2	12.6	4	1.3	26.5	3.6	8.7E-03	1.2E-01
28S ribosomal protein S23, mitochondrial	MRPS23	Q9Y3D9	10.5	21.8	2	1.3	24.5	4.3	3.6E-03	7.0E-02
Isoform PML-4 of Protein PML	PML	P29590-5	21.3	70.0	8	1.2	26.9	6.0	5.8E-04	2.8E-02
HLA class I histocompatibility antigen, B alpha chain	HLA-B	P01889	22.1	40.5	6	1.2	27.8	6.1	5.4E-04	2.8E-02
NADH dehydrogenase [ubiquinone] iron-sulfur protein 5	NDUFS5	O43920	27.4	12.5	3	1.2	25.7	3.7	8.5E-03	1.2E-01
Aldo-keto reductase family 1 member B1	AKR1B1	P15121	44.3	35.9	11	1.2	29.9	5.3	1.3E-03	3.9E-02
Anillin	ANLN	Q9NQW6	4.4	124.2	4	1.2	24.5	5.7	8.2E-04	3.2E-02
V-type proton ATPase subunit G 1	ATP6V1G1	O75348	16.9	13.8	2	1.2	25.9	3.6	8.6E-03	1.2E-01
HLA class I histocompatibility antigen, alpha chain G	HLA-G	P17693	6.8	38.2	2	1.2	26.9	4.9	2.0E-03	4.9E-02

Protein name	Gene name	uniprotID	Sequence coverage (%)	Mol_weight kDa	Nr. Peptides	log2FC	AveExpr	t	P.Value	adj.P.Val
Isoform 2 of Tropomyosin beta chain	TPM2	P07951-2	57.7	33.0	25	1.1	31.7	3.9	6.1E-03	9.6E-02
Alpha- and gamma-adaptin-binding protein p34	AAGAB	Q6PD74	23.2	34.6	4	1.1	24.6	3.9	5.9E-03	9.3E-02
Isoform 2 of Deoxyuridine 5-triphosphate nucleotidohydrolase, mitochondrial	DUT	P33316-2	32.9	17.7	4	1.1	26.1	5.1	1.5E-03	4.4E-02
Isoform 3 of Adenosine kinase	ADK	P55263-3	14.4	34.1	2	1.1	25.5	4.0	5.6E-03	9.1E-02
28S ribosomal protein S28, mitochondrial	MRPS28	Q9Y2Q9	26.2	20.8	3	1.1	24.2	5.0	1.6E-03	4.6E-02
Selenoprotein F	SELENOF	O60613	17.6	18.1	2	1.1	24.3	4.9	2.0E-03	4.9E-02
Leucine-rich repeat-containing protein 59	LRRC59	Q96AG4	29.0	34.9	9	1.1	29.2	5.4	1.2E-03	3.7E-02
Isocitrate dehydrogenase [NAD] subunit alpha, mitochondrial	IDH3A	P50213	22.1	39.6	7	1.1	27.4	5.3	1.2E-03	3.7E-02
Isoform Beta-4D of Integrin beta-4	ITGB4	P16144-4	11.7	194.5	15	1.1	27.1	5.4	1.1E-03	3.7E-02
Endonuclease domain-containing 1 protein	ENDOD1	O94919	23.0	55.0	6	1.0	25.4	4.7	2.5E-03	5.8E-02
Transgelin	TAGLN	Q01995	70.1	22.6	15	1.0	31.1	5.0	1.8E-03	4.7E-02
Myosin regulatory light chain 12A	MYL12A	P19105	72.5	19.8	13	1.0	28.1	3.3	1.5E-02	1.5E-01
Cytochrome b-c1 complex subunit 1, mitochondrial	UQCRC1	P31930	23.1	52.6	8	1.0	28.7	5.4	1.1E-03	3.7E-02
Myosin regulatory light polypeptide 9	MYL9	P24844	72.1	19.8	12	1.0	28.4	4.4	3.6E-03	7.0E-02
Annexin A6	ANXA6	P08133	39.7	75.9	24	1.0	29.7	4.4	3.5E-03	6.8E-02
Isoform p18 of 7,8-dihydro-8-oxoguanine triphosphatase	NUDT1	P36639-4	25.6	18.0	3	1.0	24.1	4.3	3.8E-03	7.2E-02
Cytochrome c	CYCS	P99999	54.3	11.7	5	1.0	27.3	3.9	6.6E-03	1.0E-01
Isoform Short of Proliferation marker protein Ki-67	MKI67	P46013-2	6.8	319.4	11	1.0	26.1	5.0	1.7E-03	4.7E-02
39S ribosomal protein L12, mitochondrial	MRPL12	P52815	40.4	21.3	4	1.0	27.8	3.2	1.6E-02	1.6E-01
Integrin beta-5	ITGB5	P18084	6.9	88.1	4	0.9	25.8	6.0	5.8E-04	2.8E-02
Cytochrome c oxidase subunit 7C, mitochondrial	COX7C	P15954	28.6	7.2	2	0.9	26.1	3.5	1.0E-02	1.3E-01
Ubiquitin-conjugating enzyme E2 C	UBE2C	O00762	38.5	19.7	4	0.9	24.6	3.0	1.9E-02	1.8E-01
Cytochrome c oxidase subunit 5A, mitochondrial	COX5A	P20674	51.3	16.8	6	0.9	29.5	3.4	1.2E-02	1.4E-01
39S ribosomal protein L13, mitochondrial	MRPL13	Q9BYD1	14.0	20.7	2	0.9	25.6	3.1	1.8E-02	1.7E-01
TP53-binding protein 1	TP53BP1	Q12888	3.6	213.6	5	0.9	25.2	3.3	1.3E-02	1.5E-01
Isoform 3 of Na(+)/H(+) exchange regulatory cofactor NHE-RF2	SLC9A3R2	Q15599-3	11.5	24.7	2	0.9	23.8	2.9	2.4E-02	1.9E-01
Isoform 4 of Branched-chain-amino-acid aminotransferase, cytosolic	BCAT1	P54687-4	22.1	42.8	6	0.9	26.9	3.2	1.6E-02	1.6E-01
Isoform 2 of ATPase family AAA domain-containing protein 3A	ATAD3A	Q9NVI7-2	9.7	66.2	5	0.9	25.8	3.8	7.0E-03	1.0E-01
Isoform 3 of Nucleoside diphosphate kinase B	NME2	P22392-2	69.7	30.1	13	0.9	31.7	5.0	1.7E-03	4.6E-02
39S ribosomal protein L38, mitochondrial	MRPL38	Q96DV4	7.9	44.6	3	0.9	25.4	5.1	1.5E-03	4.4E-02
Cytosolic Fe-S cluster assembly factor NUBP2	NUBP2	Q9Y5Y2	19.6	28.8	2	0.9	25.3	2.8	2.7E-02	2.0E-01
Ubiquitin carboxyl-terminal hydrolase isozyme L1	UCHL1	P09936	66.4	24.8	10	0.9	32.6	4.4	3.4E-03	6.8E-02
Fructose-2,6-bisphosphatase TIGAR	TIGAR	Q9NQ88	10.4	30.1	2	0.9	25.0	3.7	8.5E-03	1.2E-01
Isoform 3 of Protein O-glucosyltransferase 3	POGLUT3	Q7Z4H8-3	7.3	47.4	2	0.8	24.2	3.1	1.7E-02	1.7E-01
Phosphatidylinositol transfer protein beta isoform	PITPNB	P48739	21.8	31.5	4	0.8	25.2	2.7	3.3E-02	2.3E-01

Protein name	Gene name	uniprotID	Sequence coverage (%)	Mol_weight kDa	Nr. Peptides	log2FC	AveExpr	t	P.Value	adj.P.Val
Histone H2A type 1-J	H2AC14	Q99878	55.5	13.9	6	0.8	30.2	2.5	4.0E-02	2.5E-01
Eukaryotic translation initiation factor 3 subunit J	EIF3J	O75822	34.1	29.1	8	0.8	27.0	3.9	6.2E-03	9.6E-02
Isoform 5 of La-related protein 4	LARP4	Q71RC2-5	11.3	72.4	6	0.8	25.3	4.6	2.6E-03	5.8E-02
Hepatoma-derived growth factor-related protein 3	HDGFL3	Q9Y3E1	27.6	22.6	5	0.8	25.4	2.9	2.3E-02	1.9E-01
Calponin-2	CNN2	Q99439	29.4	33.7	7	0.8	30.6	3.2	1.5E-02	1.5E-01
Protein S100-A16	S100A16	Q96FQ6	51.5	11.8	4	0.8	27.5	3.1	1.7E-02	1.7E-01
Calcineurin B homologous protein 1	CHP1	Q99653	14.4	22.5	2	0.8	23.5	2.8	2.7E-02	2.0E-01
NADH dehydrogenase [ubiquinone] iron-sulfur protein 8, mitochondrial	NDUFS8	O00217	9.5	23.7	2	0.8	26.0	3.3	1.4E-02	1.5E-01
Coiled-coil domain-containing protein 58	CCDC58	Q4VC31	28.5	16.6	3	0.8	26.1	3.0	2.1E-02	1.8E-01
Isoform 2 of Protein Niban 2	NIBAN2	Q96TA1-2	28.1	82.7	15	0.8	28.6	4.6	2.6E-03	5.8E-02
39S ribosomal protein L23, mitochondrial	MRPL23	Q16540	23.5	17.8	2	0.8	24.4	3.0	2.0E-02	1.8E-01
Isoform 2 of Cytosolic Fe-S cluster assembly factor NUBP1	NUBP1	P53384-2	11.7	33.4	2	0.8	25.7	2.9	2.3E-02	1.9E-01
Inorganic pyrophosphatase 2, mitochondrial	PPA2	Q9H2U2	34.7	37.9	9	0.8	27.9	4.6	2.6E-03	5.8E-02
HLA class I histocompatibility antigen, C alpha chain	HLA-C	P10321	20.2	40.6	5	0.8	25.7	3.2	1.7E-02	1.6E-01
Ubiquitin carboxyl-terminal hydrolase isozyme L3	UCHL3	P15374	51.7	26.2	8	0.8	28.5	3.1	1.7E-02	1.7E-01
Cytochrome b5 type B	CYB5B	O43169	49.3	16.7	4	0.8	28.5	4.0	5.8E-03	9.3E-02
Isoform 3 of Adaptin ear-binding coat-associated protein 2	NECAP2	Q9NVZ3-3	29.7	19.4	3	0.8	25.5	3.4	1.1E-02	1.3E-01
Cytochrome c1, heme protein, mitochondrial	CYC1	P08574	8.6	35.4	3	0.8	26.5	4.9	1.9E-03	4.8E-02
Integrin alpha-5	ITGA5	P08648	11.6	114.5	7	0.8	27.7	4.4	3.3E-03	6.7E-02
Isoform 4 of Small EDRK-rich factor 2	SERF2	P84101-4	37.8	5.2	2	0.8	25.7	2.5	4.5E-02	2.6E-01
NADH dehydrogenase [ubiquinone] flavoprotein 2, mitochondrial	NDUFV2	P19404	26.5	27.4	5	0.8	26.9	3.6	9.0E-03	1.2E-01
WW domain-binding protein 2	WBP2	Q969T9	52.9	28.1	8	0.8	28.5	3.7	8.5E-03	1.2E-01
Toll-interacting protein	TOLLIP	Q9H0E2	9.9	30.3	2	0.8	25.0	3.7	8.0E-03	1.1E-01
Cytoplasmic dynein 1 light intermediate chain 1	DYNC1LI1	Q9Y6G9	12.6	56.6	5	0.7	23.8	3.5	1.0E-02	1.3E-01
Coiled-coil domain-containing protein 86	CCDC86	Q9H6F5	16.1	40.2	3	0.7	24.4	3.6	9.8E-03	1.3E-01
Cytochrome b-c1 complex subunit 7	UQCRB	P14927	30.6	13.5	3	0.7	26.3	3.0	2.0E-02	1.8E-01
DnaJ homolog subfamily A member 3, mitochondrial	DNAJA3	Q96EY1	10.6	52.5	3	0.7	25.5	3.5	1.1E-02	1.3E-01
Isoform 2 of Trafficking protein particle complex subunit 3	TRAPPC3	O43617-2	19.4	15.0	3	0.7	25.3	2.9	2.5E-02	2.0E-01
Protein mago nashi homolog 2	MAGOHB	Q96A72	27.7	17.3	3	0.7	24.1	4.2	4.3E-03	7.6E-02
Corrinoid adenosyltransferase	MMAB	Q96EY8	15.2	27.4	2	0.7	24.1	2.6	3.7E-02	2.4E-01
Peptidyl-prolyl cis-trans isomerase F, mitochondrial	PPIF	P30405	23.2	22.0	4	0.7	26.5	4.2	4.4E-03	7.8E-02
Vacuolar protein-sorting-associated protein 25	VPS25	Q9BRG1	30.7	20.7	3	0.7	24.9	2.8	2.7E-02	2.0E-01
Protein disulfide-isomerase A3	PDIA3	P30101	57.6	56.8	27	0.7	32.7	3.3	1.3E-02	1.5E-01
Isoform Non-brain of Clathrin light chain B	CLTB	P09497-2	24.6	23.2	7	0.7	27.9	2.6	3.8E-02	2.4E-01
Biliverdin reductase A	BLVRA	P53004	21.6	33.4	5	0.7	25.6	3.4	1.1E-02	1.3E-01

Protein name	Gene name	uniprotID	Sequence coverage (%)	Mol_weight kDa	Nr. Peptides	log2FC	AveExpr	t	P.Value	adj.P.Val
RNA transcription, translation and transport factor protein	RTRAF	Q9Y224	45.9	28.1	10	0.7	28.8	4.0	5.8E-03	9.2E-02
Activating signal cointegrator 1 complex subunit 2	ASCC2	Q9H1I8	8.3	86.4	3	0.7	24.4	3.1	1.9E-02	1.7E-01
Inactive C-alpha-formylglycine-generating enzyme 2	SUMF2	Q8NBJ7	33.9	33.8	10	0.7	28.2	2.8	2.6E-02	2.0E-01
Histidine triad nucleotide-binding protein 2, mitochondrial	HINT2	Q9BX68	27.0	17.2	3	0.7	26.7	2.5	4.4E-02	2.6E-01
Phosducin-like protein 3	PDCL3	Q9H2J4	25.1	27.6	5	0.7	25.8	2.8	2.7E-02	2.0E-01
26S proteasome non-ATPase regulatory subunit 9	PSMD9	O00233	22.4	24.7	5	0.7	27.6	2.9	2.3E-02	1.9E-01
Isoform 3 of Palladin	PALLD	Q8WX93-3	28.1	108.6	23	0.7	30.0	4.3	3.9E-03	7.2E-02
Translationally-controlled tumor protein	TPT1	P13693	48.3	19.6	8	0.7	30.3	2.5	3.9E-02	2.5E-01
Isoform BIN1-10-13 of Myc box-dependent-interacting protein 1	BIN1	O00499-9	37.4	45.6	10	0.7	27.4	2.5	4.3E-02	2.6E-01
Actin, alpha cardiac muscle 1	ACTC1	P68032	50.9	42.0	18	0.7	34.3	4.2	4.2E-03	7.6E-02
Proteasome subunit beta type-3	PSMB3	P49720	23.4	22.9	3	0.7	25.3	4.3	3.9E-03	7.2E-02
Isoform 5 of Tropomyosin alpha-3 chain	TPM3	P06753-5	63.3	28.9	24	0.7	27.9	2.7	3.3E-02	2.3E-01
Serum paraoxonase/arylesterase 2	PON2	Q15165	39.8	39.4	6	0.7	27.9	3.0	2.0E-02	1.8E-01
D-aminoacyl-tRNA deacylase 1	DTD1	Q8TEA8	14.8	23.4	2	0.7	24.2	2.8	2.7E-02	2.0E-01
Isoform 3 of Epidermal growth factor receptor kinase substrate 8-like protein 2	EPS8L2	Q9H6S3-3	14.1	82.3	6	0.7	26.2	3.0	2.0E-02	1.8E-01
N-terminal Xaa-Pro-Lys N-methyltransferase 1	NTMT1	Q9BV86	11.2	25.4	2	0.7	25.6	3.4	1.2E-02	1.4E-01
Tight junction protein ZO-1	TJP1	Q07157	16.1	195.5	20	0.7	27.9	3.7	8.5E-03	1.2E-01
Cytochrome c oxidase subunit 7A2, mitochondrial	COX7A2	P14406	56.6	9.4	3	0.7	27.0	3.1	1.8E-02	1.7E-01
Cilia- and flagella-associated protein 36	CFAP36	Q96G28	16.7	39.4	3	0.7	24.7	2.5	4.2E-02	2.6E-01
Nicotinamide phosphoribosyltransferase	NAMPT	P43490	35.2	55.5	13	0.7	28.5	4.1	5.0E-03	8.5E-02
Costars family protein ABRACL	ABRACL	Q9P1F3	49.4	9.1	2	0.7	28.6	3.0	2.1E-02	1.8E-01
Nuclear protein localization protein 4 homolog	NPLOC4	Q8TAT6	28.3	68.1	13	0.7	27.6	4.3	3.9E-03	7.2E-02
Membrane-associated progesterone receptor component 1	PGRMC1	O00264	45.6	21.7	7	0.7	28.6	2.9	2.4E-02	2.0E-01
HLA class I histocompatibility antigen, A alpha chain	HLA-A	P04439	31.5	40.8	9	0.7	29.8	3.2	1.6E-02	1.6E-01
Adenylate kinase isoenzyme 1	AK1	P00568	40.7	21.6	7	0.7	27.8	2.4	4.7E-02	2.7E-01
E3 ubiquitin-protein ligase CHIP	STUB1	Q9UNE7	18.2	34.9	5	0.7	26.4	3.3	1.3E-02	1.5E-01
39S ribosomal protein L49, mitochondrial	MRPL49	Q13405	28.3	19.2	4	0.6	25.9	2.4	4.7E-02	2.7E-01
Cytochrome b-c1 complex subunit Rieske, mitochondrial	UQCRFS1	P47985	13.9	29.7	3	0.6	26.2	2.9	2.3E-02	1.9E-01
Pyridoxine-5-phosphate oxidase	PNPO	Q9NVS9	30.3	30.0	7	0.6	29.0	2.9	2.3E-02	1.9E-01
Isoform 2 of Ubiquilin-1	UBQLN1	Q9UMX0-2	29.8	59.2	10	0.6	27.9	2.5	4.1E-02	2.5E-01
Isoform 2 of Interferon regulatory factor 2-binding protein 2	IRF2BP2	Q7Z5L9-2	8.4	59.5	2	0.6	25.2	2.5	4.3E-02	2.6E-01
Isoform 5 of Peroxisomal biogenesis factor 19	PEX19	P40855-5	26.4	29.3	5	0.6	26.0	3.4	1.1E-02	1.3E-01
Growth factor receptor-bound protein 2	GRB2	P62993	30.0	25.2	5	0.6	26.0	3.2	1.6E-02	1.6E-01
5-nucleotidase	NT5E	P21589	44.3	63.4	18	0.6	29.8	3.8	7.4E-03	1.1E-01
Isoform Cytoplasmic of Phospholipid hydroperoxide glutathione peroxidase	GPX4	P36969-2	13.5	19.5	2	0.6	24.5	3.0	2.0E-02	1.8E-01

Protein name	Gene name	uniprotID	Sequence coverage (%)	Mol_weight kDa	Nr. Peptides	log2FC	AveExpr	t	P.Value	adj.P.Val
Tryptophan--tRNA ligase, cytoplasmic	WARS1	P23381	29.7	53.2	10	0.6	27.4	2.7	3.3E-02	2.3E-01
Mitochondrial import receptor subunit TOM40 homolog	TOMM40	O96008	13.6	37.9	2	0.6	25.7	3.2	1.5E-02	1.5E-01
Prohibitin-2	PHB2	Q99623	36.8	33.3	10	0.6	27.2	2.4	5.0E-02	2.7E-01
Interferon-induced transmembrane protein 3	IFITM3	Q01628	30.8	14.6	2	0.6	26.1	3.0	2.0E-02	1.8E-01
Mitochondrial import inner membrane translocase subunit Tim9	TIMM9	Q9Y5J7	25.8	10.4	2	0.6	26.0	2.6	3.4E-02	2.3E-01
Transcription factor A, mitochondrial	TFAM	Q00059	22.8	29.1	6	0.6	26.2	2.8	2.8E-02	2.1E-01
Mesencephalic astrocyte-derived neurotrophic factor	MANF	P55145	28.0	20.7	5	0.6	28.2	3.1	1.9E-02	1.8E-01
NADH dehydrogenase [ubiquinone] iron-sulfur protein 3, mitochondrial	NDUFS3	O75489	21.2	30.2	4	0.6	25.7	3.5	1.0E-02	1.3E-01
Prostaglandin E synthase 2	PTGES2	Q9H7Z7	15.6	41.9	4	0.6	25.9	2.9	2.4E-02	2.0E-01
Isoform 2 of SRA stem-loop-interacting RNA-binding protein, mitochondrial	SLIRP	Q9GZT3-2	53.3	12.1	5	0.6	27.8	2.5	4.1E-02	2.5E-01
Stress-70 protein, mitochondrial	HSPA9	P38646	61.0	73.7	33	0.6	31.9	2.8	2.7E-02	2.0E-01
Stomatin-like protein 2, mitochondrial	STOML2	Q9UJZ1	40.2	38.5	10	0.6	28.1	2.7	3.1E-02	2.2E-01
DBIRD complex subunit ZNF326	ZNF326	Q5BKZ1	10.1	65.7	4	-0.6	25.4	-3.6	9.3E-03	1.2E-01
Protein canopy homolog 3	CNPY3	Q9BT09	9.7	30.7	2	-0.6	25.1	-2.7	3.1E-02	2.2E-01
COP9 signalosome complex subunit 4	COPS4	Q9BT78	25.6	46.3	7	-0.6	26.0	-2.5	4.0E-02	2.5E-01
Isoform 2 of Myosin phosphatase Rho-interacting protein	MPRIP	Q6WCQ1-2	35.9	118.1	28	-0.6	28.2	-3.3	1.4E-02	1.5E-01
Isoform 3 of Pleckstrin homology-like domain family B member 1	PHLDB1	Q86UU1-3	12.0	107.4	10	-0.6	26.2	-3.4	1.2E-02	1.4E-01
Zinc finger RNA-binding protein	ZFR	Q96KR1	4.6	117.0	3	-0.6	23.9	-2.8	2.7E-02	2.0E-01
Isoform 3 of DNA repair protein RAD50	RAD50	Q92878-3	10.9	138.4	13	-0.6	26.3	-3.3	1.3E-02	1.5E-01
Pyridoxal kinase	PDXK	O00764	36.5	35.1	8	-0.6	26.9	-3.2	1.6E-02	1.6E-01
Isoform 2 of Elongation factor-like GTPase 1	EFL1	Q7Z2Z2-2	8.2	119.9	5	-0.6	25.6	-3.6	9.1E-03	1.2E-01
Isoform 2 of MAGUK p55 subfamily member 5	MPP5	Q8N3R9-2	25.6	73.4	10	-0.7	26.3	-2.9	2.3E-02	1.9E-01
Isoform 2 of Phytanoyl-CoA dioxygenase domain-containing protein 1	PHYHD1	Q5SRE7-2	20.7	30.0	4	-0.7	25.1	-3.7	8.6E-03	1.2E-01
Isoform 5 of Afadin	AFDN	P55196-5	17.7	207.8	26	-0.7	27.8	-3.8	6.8E-03	1.0E-01
Isoform Beta of Caspase-7	CASP7	P55210-2	6.3	28.0	2	-0.7	26.8	-3.0	2.1E-02	1.8E-01
Isoform 2 of 4F2 cell-surface antigen heavy chain	SLC3A2	P08195-2	45.0	57.9	17	-0.7	29.6	-3.8	7.5E-03	1.1E-01
Isoform 2 of Haloacid dehalogenase-like hydrolase domain-containing protein 2	HDHD2	Q9H0R4-2	20.1	18.5	2	-0.7	24.2	-3.9	6.3E-03	9.7E-02
ADP-ribose glycohydrolase MACROD1	MACROD1	Q9BQ69	10.2	35.5	2	-0.7	24.4	-2.5	4.3E-02	2.6E-01
Isoform 3 of Septin-10	SEPTIN10	Q9P0V9-3	15.5	50.1	5	-0.7	25.6	-3.3	1.4E-02	1.5E-01
Isoform 2 of Annexin A11	ANXA11	P50995-2	27.8	51.2	14	-0.7	28.9	-2.4	4.7E-02	2.7E-01
Isoform 3 of Slit homolog 3 protein	SLIT3	O75094-3	7.8	162.2	10	-0.7	27.8	-3.3	1.3E-02	1.5E-01
Filamin-C	FLNC	Q14315	58.0	291.0	124	-0.7	34.5	-4.1	4.8E-03	8.1E-02
Isoform Soluble of Catechol O-methyltransferase	COMT	P21964-2	14.5	24.4	3	-0.7	25.7	-2.4	4.8E-02	2.7E-01
Isoform 4 of Ankycorbin	RAI14	Q9P0K7-4	29.4	106.9	25	-0.7	28.5	-3.5	1.0E-02	1.3E-01
Isoform 2 of Uveal autoantigen with coiled-coil domains and ankyrin repeats	UACA	Q9BZF9-2	21.0	161.5	25	-0.7	27.7	-3.5	1.0E-02	1.3E-01

Protein name	Gene name	uniprotID	Sequence coverage (%)	Mol_weight kDa	Nr. Peptides	log2FC	AveExpr	t	P.Value	adj.P.Val
Aspartyl aminopeptidase	DNPEP	Q9ULA0	9.3	52.4	2	-0.7	24.1	-3.5	1.1E-02	1.3E-01
Isoform 5 of Inactive tyrosine-protein kinase 7	PTK7	Q13308-5	12.0	89.8	6	-0.7	25.3	-3.1	1.7E-02	1.7E-01
Lamin-B1	LMNB1	P20700	57.7	66.4	34	-0.7	30.2	-4.2	4.5E-03	7.9E-02
RNA-binding protein 12	RBM12	Q9NTZ6	6.8	97.4	5	-0.7	25.2	-3.3	1.3E-02	1.5E-01
Protein-lysine 6-oxidase	LOX	P28300	17.5	46.9	4	-0.7	24.3	-2.5	4.2E-02	2.6E-01
Phosphoglycerate kinase 1	PGK1	P00558	60.4	44.6	22	-0.7	33.2	-4.1	4.6E-03	8.0E-02
Isoform 3 of Microtubule-associated protein 2	MAP2	P11137-3	7.0	199.1	9	-0.7	26.2	-4.0	5.6E-03	9.1E-02
Prolyl endopeptidase	PREP	P48147	8.9	80.7	6	-0.7	25.0	-3.3	1.4E-02	1.5E-01
Rho-associated protein kinase 2	ROCK2	O75116	4.7	160.9	5	-0.7	24.2	-2.5	4.4E-02	2.6E-01
Utrophin	UTRN	P46939	10.5	394.5	29	-0.7	27.6	-4.0	5.3E-03	8.8E-02
Band 4.1-like protein 2	EPB41L2	O43491	26.8	112.6	17	-0.7	27.6	-4.8	2.2E-03	5.2E-02
Radixin	RDX	P35241	38.4	68.6	24	-0.8	28.2	-4.7	2.3E-03	5.5E-02
Isoform 2 of Syntaxin-7	STX7	O15400-2	34.3	27.4	5	-0.8	25.6	-2.5	4.4E-02	2.6E-01
Protein disulfide-isomerase A5	PDIA5	Q14554	15.4	59.6	5	-0.8	26.2	-2.9	2.5E-02	2.0E-01
Ubiquitin-like modifier-activating enzyme 6	UBA6	A0AVT1	10.3	118.0	8	-0.8	26.0	-3.0	2.0E-02	1.8E-01
Isoform 4 of Myosin light chain kinase, smooth muscle	MYLK	Q15746-5	20.9	202.4	31	-0.8	28.7	-4.6	2.6E-03	5.8E-02
Isoform 2 of Cleavage and polyadenylation specificity factor subunit 7	CPSF7	Q8N684-2	16.9	51.1	6	-0.8	25.1	-2.5	4.0E-02	2.5E-01
Isoform 2 of Sodium/potassium-transporting ATPase subunit beta-1	ATP1B1	P05026-2	15.6	34.9	3	-0.8	26.4	-2.7	3.4E-02	2.3E-01
Isoform L-APP733 of Amyloid-beta precursor protein	APP	P05067-7	5.5	82.9	4	-0.8	25.3	-4.2	4.5E-03	7.8E-02
CD59 glycoprotein	CD59	P13987	15.6	14.2	2	-0.8	27.5	-2.5	4.2E-02	2.6E-01
Isoform 3 of Brain-specific angiogenesis inhibitor 1-associated protein 2	BAIAP2	Q9UQB8-3	39.3	56.6	17	-0.8	27.9	-3.6	9.6E-03	1.3E-01
Isoform 10 of LIM and calponin homology domains-containing protein 1	LIMCH1	Q9UPQ0-10	38.3	119.0	34	-0.8	29.6	-3.7	7.9E-03	1.1E-01
Axin interactor, dorsalization-associated protein	AIDA	Q96BJ3	20.3	35.0	4	-0.8	22.8	-3.2	1.6E-02	1.6E-01
Heat shock 70 kDa protein 6	HSPA6	P17066	13.5	71.0	8	-0.8	29.7	-3.8	7.4E-03	1.1E-01
V-type proton ATPase subunit D	ATP6V1D	Q9Y5K8	15.8	28.3	2	-0.8	23.1	-3.5	1.0E-02	1.3E-01
Interleukin-18	IL18	Q14116	39.4	22.3	8	-0.8	29.0	-3.9	6.2E-03	9.6E-02
Dihydropyrimidinase-related protein 4	DPYSL4	O14531	14.9	61.9	5	-0.9	24.9	-3.4	1.2E-02	1.4E-01
High mobility group protein B2	HMGB2	P26583	18.2	24.0	4	-0.9	29.5	-3.5	1.1E-02	1.3E-01
Isoform 2 of Small nuclear ribonucleoprotein Sm D3	SNRPD3	P62318-2	15.8	13.3	2	-0.9	25.8	-3.2	1.5E-02	1.6E-01
Isoform 6 of Rho GTPase-activating protein 17	ARHGAP17	Q68EM7-6	11.7	90.9	6	-0.9	25.4	-5.5	1.0E-03	3.7E-02
CBP80/20-dependent translation initiation factor	CTIF	O43310	5.2	67.6	3	-0.9	24.0	-3.5	1.1E-02	1.3E-01
Isoform 12 of Intersectin-1	ITSN1	Q15811-12	6.1	125.9	5	-0.9	24.4	-2.8	2.8E-02	2.0E-01
Isoform 2 of Procollagen-lysine,2-oxoglutarate 5-dioxygenase 2	PLOD2	O00469-2	29.9	87.1	21	-0.9	29.1	-4.5	2.8E-03	6.0E-02
1-phosphatidylinositol 4,5-bisphosphate phosphodiesterase beta-4	PLCB4	Q15147	25.3	134.5	26	-0.9	29.2	-5.7	8.5E-04	3.3E-02
Isoform Delta 8 of Calcium/calmodulin-dependent protein kinase type II subunit delta	CAMK2D	Q13557-5	19.1	56.2	6	-0.9	25.4	-4.0	5.3E-03	8.8E-02

Protein name	Gene name	uniprotID	Sequence coverage (%)	Mol_weight kDa	Nr. Peptides	log2FC	AveExpr	t	P.Value	adj.P.Val
EH domain-containing protein 1	EHD1	Q9H4M9	59.0	60.6	23	-0.9	29.3	-4.3	3.9E-03	7.2E-02
Isoform 4 of Neurofascin	NFASC	O94856-4	10.5	119.5	7	-0.9	25.9	-4.6	2.7E-03	5.8E-02
m7GpppX diphosphatase	DCPS	Q96C86	35.0	38.6	6	-0.9	25.0	-3.8	6.9E-03	1.0E-01
Isoform 2 of FK506-binding protein 15	FKBP15	Q5T1M5-2	5.6	132.5	5	-0.9	24.3	-4.0	5.7E-03	9.1E-02
Laminin subunit gamma-1	LAMC1	P11047	13.4	177.6	15	-0.9	26.7	-3.3	1.3E-02	1.5E-01
1,2-dihydroxy-3-keto-5-methylthiopentene dioxygenase	ADI1	Q9BV57	53.1	21.5	7	-0.9	27.7	-4.6	2.5E-03	5.8E-02
Isoform 7 of Coxsackievirus and adenovirus receptor	CXADR	P78310-7	24.1	35.6	6	-0.9	27.2	-4.4	3.4E-03	6.8E-02
Galectin-3	LGALS3	P17931	28.0	26.2	7	-0.9	28.9	-4.3	3.6E-03	7.0E-02
Ankyrin repeat domain-containing protein 13A	ANKRD13A	Q8IZ07	19.7	67.6	7	-1.0	25.3	-2.9	2.5E-02	2.0E-01
Bifunctional 3-phosphoadenosine 5-phosphosulfate synthase 1	PAPSS1	O43252	9.3	70.8	5	-1.0	26.1	-2.5	4.5E-02	2.6E-01
ERO1-like protein alpha	ERO1A	Q96HE7	23.1	54.4	9	-1.0	26.4	-3.1	1.8E-02	1.7E-01
Isoform UBF2 of Nucleolar transcription factor 1	UBTF	P17480-2	7.0	84.9	4	-1.0	24.6	-2.9	2.5E-02	2.0E-01
Isoform 2 of GDNF family receptor alpha-1	GFRA1	P56159-2	5.2	50.8	2	-1.0	26.2	-3.1	1.7E-02	1.7E-01
NEDD8-conjugating enzyme Ubc12	UBE2M	P61081	37.7	20.9	5	-1.0	26.4	-5.4	1.1E-03	3.7E-02
Caveolin-1	CAV1	Q03135	34.3	20.5	5	-1.1	27.6	-2.4	4.9E-02	2.7E-01
Isoform 6 of Agrin	AGRN	O00468-6	6.9	214.8	11	-1.1	26.6	-6.0	6.2E-04	2.8E-02
Phosphoglucomutase-1	PGM1	P36871	26.5	61.4	11	-1.1	26.3	-2.8	2.7E-02	2.0E-01
Coiled-coil domain-containing protein 85C	CCDC85C	A6NKD9	22.2	45.2	7	-1.1	25.8	-5.8	7.5E-04	3.0E-02
Inhibitor of nuclear factor kappa-B kinase-interacting protein	IKBIP	Q70UQ0	16.0	39.3	4	-1.2	24.2	-6.1	5.7E-04	2.8E-02
Rho GTPase-activating protein 35	ARHGAP35	Q9NRY4	6.7	170.5	7	-1.2	25.4	-6.3	4.5E-04	2.6E-02
Collagen alpha-1(XII) chain	COL12A1	Q99715	18.8	333.1	36	-1.2	27.8	-6.6	3.3E-04	2.4E-02
Isoform 3 of Collagen alpha-1(XVIII) chain	COL18A1	P39060-2	9.1	135.8	6	-1.2	25.5	-4.4	3.3E-03	6.7E-02
Isoform ZB of Plasma membrane calcium-transporting ATPase 4	ATP2B4	P23634-7	14.3	132.6	12	-1.2	26.7	-2.4	4.8E-02	2.7E-01
Ras-related protein Rab-9A	RAB9A	P51151	39.3	22.8	6	-1.2	25.6	-4.3	4.0E-03	7.4E-02
Isoform 1 of TRIO and F-actin-binding protein	TRIOBP	Q9H2D6-5	19.9	68.0	11	-1.2	26.8	-6.9	2.7E-04	2.3E-02
Protein phosphatase methylesterase 1	PPME1	Q9Y570	24.9	42.3	7	-1.2	26.0	-5.3	1.2E-03	3.7E-02
Isoform 2 of Apoptosis-stimulating of p53 protein 2	TP53BP2	Q13625-2	13.7	111.4	8	-1.2	25.2	-5.9	6.4E-04	2.8E-02
Flotillin-2	FLOT2	Q14254	23.8	47.1	8	-1.2	26.6	-4.9	2.0E-03	4.9E-02
Dystrophin	DMD	P11532	2.0	426.7	6	-1.2	24.5	-7.5	1.6E-04	1.8E-02
Isoform 3 of Leucine zipper transcription factor-like protein 1	LZTFL1	Q9NQ48-3	26.2	29.3	5	-1.2	25.0	-3.0	2.0E-02	1.8E-01
Tissue factor	F3	P13726	17.3	33.1	5	-1.2	28.1	-5.6	8.7E-04	3.3E-02
Glypican-1	GPC1	P35052	21.5	61.7	9	-1.2	27.1	-6.6	3.4E-04	2.4E-02
Isoform 3 of Matrilin-2	MATN2	O00339-3	10.6	102.2	8	-1.3	26.3	-4.1	4.6E-03	7.9E-02
Caveolae-associated protein 1	CAVIN1	Q6NZI2	15.9	43.5	5	-1.3	26.8	-7.1	2.2E-04	2.1E-02
Isoform 5 of TOM1-like protein 2	TOM1L2	Q6ZVM7-5	12.0	53.4	4	-1.3	24.9	-5.5	1.0E-03	3.6E-02

Protein name	Gene name	uniprotID	Sequence coverage (%)	Mol_weight kDa	Nr. Peptides	log2FC	AveExpr	t	P.Value	adj.P.Val
TRAF2 and NCK-interacting protein kinase	TNIK	Q9UKE5	4.4	154.9	5	-1.3	23.9	-5.0	1.7E-03	4.6E-02
Isoform 2 of Carboxypeptidase D	CPD	O75976-2	5.1	126.5	4	-1.3	24.9	-3.8	7.3E-03	1.1E-01
Isoform 5 of Amyloid-like protein 2	APLP2	Q06481-5	11.7	59.2	5	-1.4	24.9	-6.0	5.8E-04	2.8E-02
Glucose-6-phosphate isomerase	GPI	P06744	52.0	63.1	22	-1.4	30.7	-3.3	1.3E-02	1.5E-01
Phosphoglucomutase-like protein 5	PGM5	Q15124	31.0	62.2	15	-1.5	27.1	-2.7	3.0E-02	2.2E-01
RNA-binding protein 25	RBM25	P49756	10.6	100.2	6	-1.5	26.0	-4.9	1.9E-03	4.9E-02
Laminin subunit beta-2	LAMB2	P55268	16.0	196.0	22	-1.5	27.8	-7.0	2.4E-04	2.2E-02
Isoform 3 of Afadin	AFDN	P55196-3	18.2	197.7	25	-1.5	23.5	-6.3	4.4E-04	2.5E-02
DCC-interacting protein 13-alpha	APPL1	Q9UKG1	6.1	79.7	3	-1.6	23.7	-5.1	1.6E-03	4.6E-02
Isoform 6 of Unconventional myosin-VI	MYO6	Q9UM54-6	6.5	148.7	4	-1.6	22.2	-2.7	3.1E-02	2.2E-01
Flotillin-1	FLOT1	O75955	27.6	47.4	9	-1.6	25.6	-9.9	2.8E-05	6.0E-03
Protein RCC2	RCC2	Q9P258	8.6	56.1	3	-1.6	23.7	-3.7	8.3E-03	1.2E-01
Protein S100-A10	S100A10	P60903	17.5	11.2	2	-1.7	29.5	-6.4	4.2E-04	2.5E-02
Junction plakoglobin	JUP	P14923	10.5	81.7	6	-1.7	24.9	-5.7	8.1E-04	3.2E-02
Isoform 5 of CUGBP Elav-like family member 2	CELF2	O95319-5	13.3	52.1	3	-1.7	25.4	-6.6	3.4E-04	2.4E-02
Isoform 2 of Programmed cell death protein 4	PDCD4	Q53EL6-2	22.7	50.6	7	-1.7	26.2	-8.5	7.1E-05	1.1E-02
Uncharacterized protein C6orf132	C6orf132	Q5T0Z8	5.5	124.0	5	-1.7	23.8	-6.4	4.0E-04	2.5E-02
Isoform C of Collagen alpha-1(XI) chain	COL11A1	P12107-3	11.0	176.6	14	-1.8	26.4	-11.7	9.3E-06	2.8E-03
Isoform A of Chloride intracellular channel protein 6	CLIC6	Q96NY7-2	41.3	71.0	16	-1.8	28.5	-6.1	5.6E-04	2.8E-02
SPARC	SPARC	P09486	22.1	34.6	4	-1.8	28.0	-5.8	7.5E-04	3.0E-02
Ribosomal protein S6 kinase alpha-3	RPS6KA3	P51812	8.8	83.7	5	-1.8	23.7	-2.5	4.1E-02	2.5E-01
Aldehyde dehydrogenase family 1 member A3	ALDH1A3	P47895	35.7	56.1	17	-1.8	28.9	-8.7	6.3E-05	1.1E-02
EH domain-containing protein 2	EHD2	Q9NZN4	15.1	61.2	7	-1.8	25.3	-4.0	5.2E-03	8.8E-02
Bifunctional 3-phosphoadenosine 5-phosphosulfate synthase 2	PAPSS2	O95340	42.3	69.5	21	-1.9	30.3	-8.3	8.3E-05	1.2E-02
Dickkopf-related protein 1	DKK1	O94907	12.4	28.7	2	-1.9	25.2	-6.5	3.7E-04	2.4E-02
Pro-cathepsin H	CTSH	P09668	10.4	37.4	3	-1.9	27.5	-9.8	2.9E-05	6.0E-03
Creatine kinase B-type	CKB	P12277	13.1	42.6	4	-1.9	23.3	-5.8	7.4E-04	3.0E-02
Isoform 2 of SH2 domain-containing protein 4A	SH2D4A	Q9H788-2	26.7	47.0	7	-1.9	26.9	-8.0	1.0E-04	1.4E-02
Isoform 4 of Multiple PDZ domain protein	MPDZ	O75970-5	7.7	214.8	10	-2.0	26.1	-4.7	2.4E-03	5.8E-02
1-phosphatidylinositol 4,5-bisphosphate phosphodiesterase delta-3	PLCD3	Q8N3E9	16.0	89.3	8	-2.0	24.2	-5.0	1.6E-03	4.6E-02
Isoform 1 of Four and a half LIM domains protein 1	FHL1	Q13642-1	11.1	31.9	3	-2.0	25.9	-7.9	1.2E-04	1.4E-02
Isoform 2 of Liprin-alpha-1	PPFIA1	Q13136-2	4.1	134.0	3	-2.0	23.1	-2.6	3.4E-02	2.3E-01
Isoform 3 of KN motif and ankyrin repeat domain-containing protein 2	KANK2	Q63ZY3-3	9.5	90.0	6	-2.0	23.8	-6.0	6.2E-04	2.8E-02
Transforming growth factor-beta-induced protein ig-h3	TGFBI	Q15582	44.4	74.7	23	-2.1	29.9	-9.2	4.5E-05	8.6E-03
Isoform 2 of Pre-mRNA-processing factor 6	PRPF6	O94906-2	5.8	102.4	4	-2.2	23.4	-2.4	4.9E-02	2.7E-01

Protein name	Gene name	uniprotID	Sequence coverage (%)	Mol_weight kDa	Nr. Peptides	log2FC	AveExpr	t	P.Value	adj.P.Val
Leucine-rich repeat transmembrane protein FLRT2	FLRT2	O43155	12.9	74.0	5	-2.6	24.5	-12.0	8.0E-06	2.8E-03
Plasminogen activator inhibitor 2	SERPINB2	P05120	17.8	46.6	5	-2.7	23.5	-5.4	1.1E-03	3.7E-02
Alpha-N-acetylgalactosaminidase	NAGA	P17050	14.6	46.6	4	-2.7	23.4	-3.0	2.2E-02	1.8E-01
Protein crumbs homolog 2	CRB2	Q5IJ48	8.0	134.3	7	-2.8	24.2	-5.9	6.5E-04	2.8E-02
Isoform 2 of Laminin subunit alpha-4	LAMA4	Q16363-2	5.6	201.8	8	-2.9	24.5	-2.7	3.2E-02	2.3E-01
Periplakin	PPL	O60437	27.3	204.7	39	-3.0	27.7	-16.8	8.6E-07	5.0E-04
Mth938 domain-containing protein	AAMDC	Q9H7C9	17.2	13.3	2	-3.1	22.9	-16.5	9.6E-07	5.0E-04
Vasorin	VASN	Q6EMK4	19.6	71.7	7	-3.2	24.3	-4.4	3.4E-03	6.8E-02
Protein NDRG1	NDRG1	Q92597	30.7	42.8	7	-3.2	26.8	-14.8	2.0E-06	8.5E-04
Sulfotransferase 1A1	SULT1A1	P50225	24.1	34.2	5	-3.7	23.9	-5.1	1.5E-03	4.4E-02
Isoform 2 of Nebulette	NEBL	O76041-2	32.2	31.2	7	-4.4	23.9	-6.3	4.7E-04	2.6E-02
Carbonic anhydrase 9	CA9	Q16790	22.0	49.7	7	-5.2	24.8	-23.0	1.1E-07	2.2E-04
Melanotransferrin	MELTF	P08582	27.9	80.2	14	-5.9	25.8	-17.0	8.0E-07	5.0E-04

Supplementary figure S1

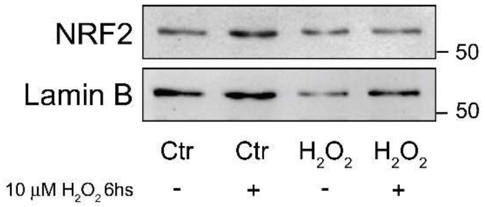

Figure S1. Assessment of Nrf2 nuclear translocation by western blotting. Ctr and H2O2-treated cells were exposed to 10µM H_2O_2 for 6h before harvesting.

Supplementary figure S2

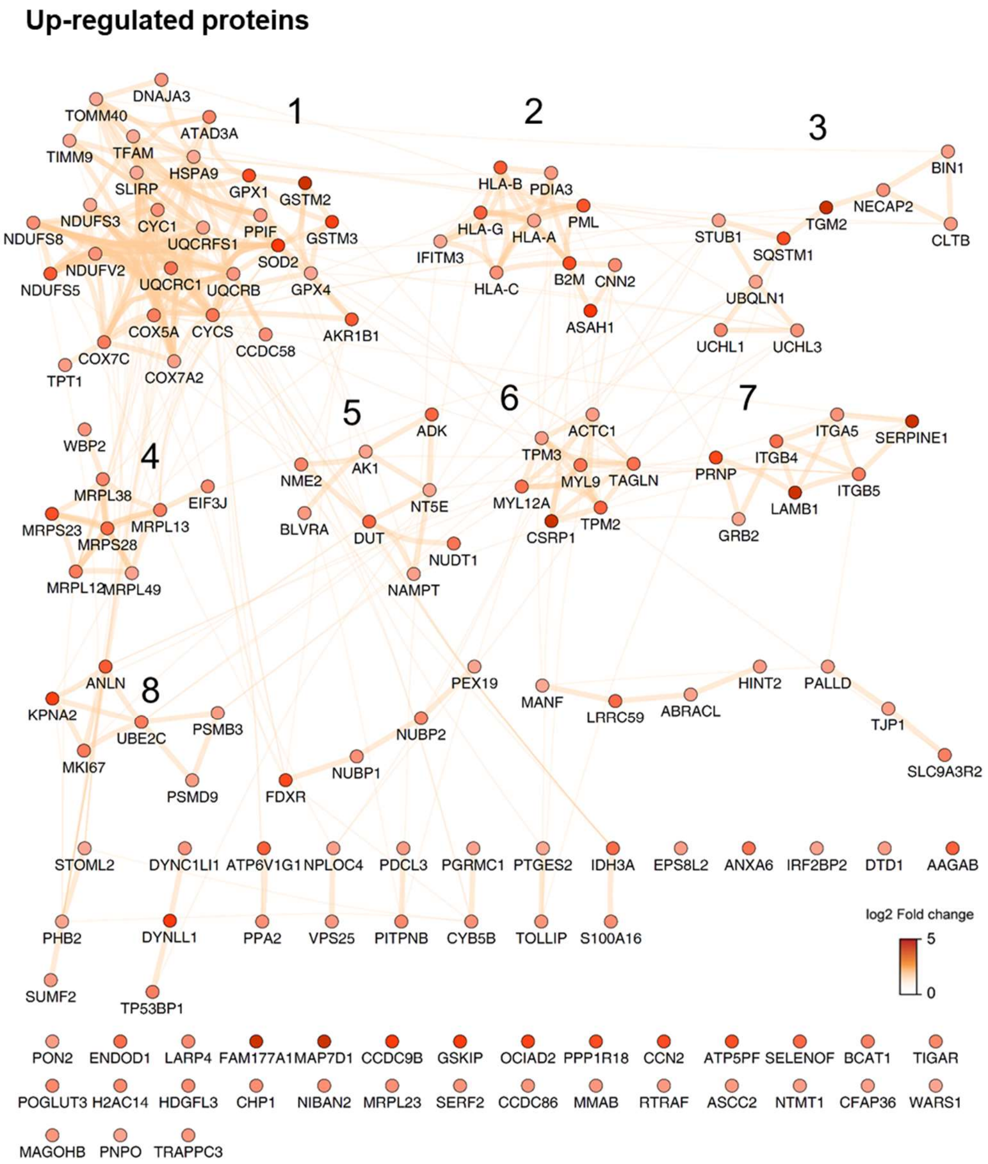

1. Oxidative phosphorylation, electron transport, hydroxide/glutathione metabolism
2. Antigen presentation, immune response
3. Vesicle-mediated transport, endocytosis, protein catabolic process
4. Mitochondrial translation
5. Nucleoside/nucleotide metabolic process
6. Muscle contraction
7. Integrin signaling
8. Cell cycle process

Network analysis of up-regulated proteins in H_2O_2-treated ARPE-19 cells. Protein interaction networks were download from STRING database and visualised with Cytoscape. The network was clustered according to the strength of interaction (combined score from STRING) and each cluster was analysed for functional enrichment of biological processes. Color of the nodes is relative to the log2 fold change of protein expression H_2O_2 vs Ctr (non-treated). The width of the edges is proportional to the strength of the interaction. Eight clusters showed significant association with specific biological process, which are reported below the figure with the corresponding number.

Supplementary figure S3

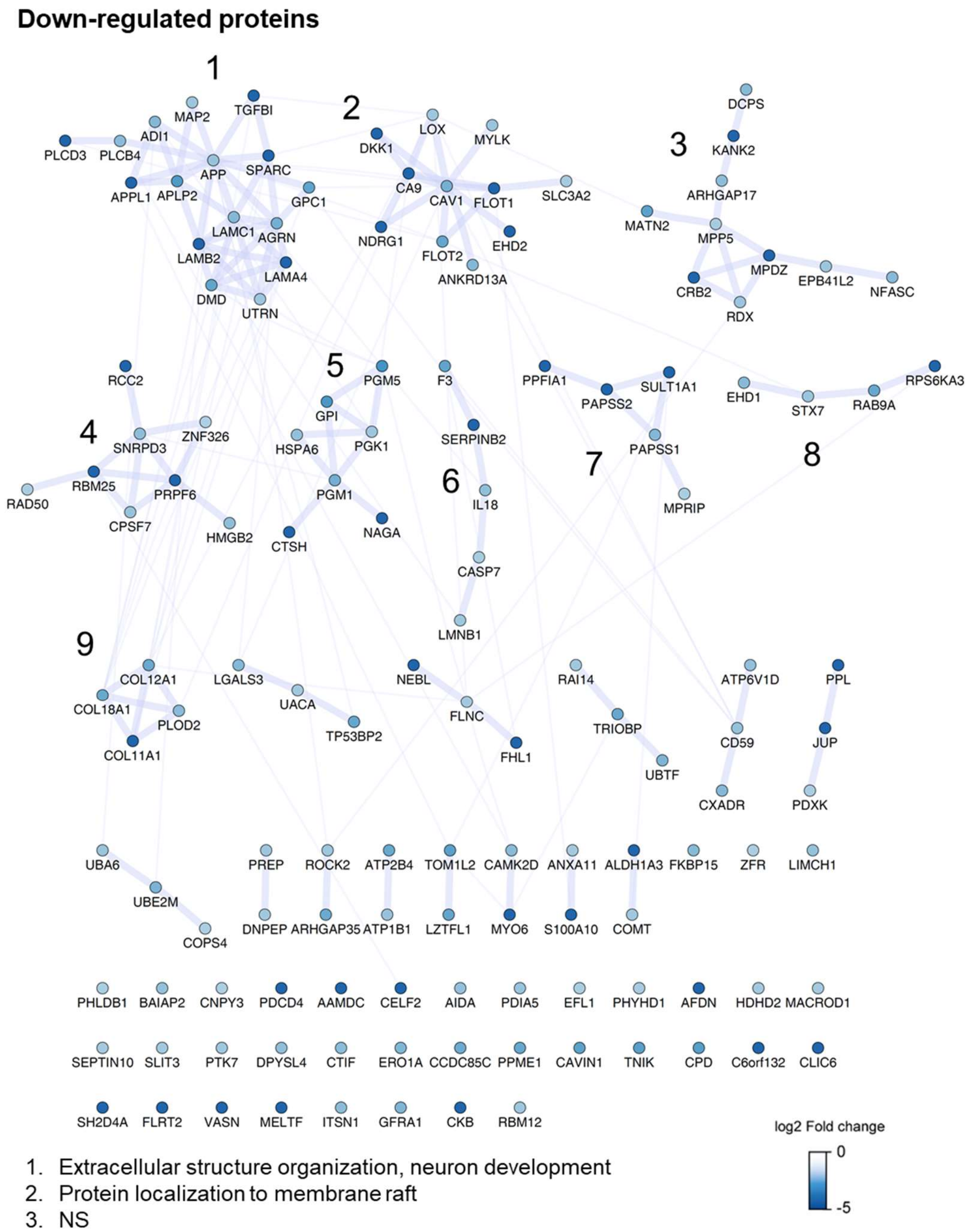

1. Extracellular structure organization, neuron development
2. Protein localization to membrane raft
3. NS
4. RNA splicing
5. Glucose metabolism
6. NS
7. Sulfate metabolic process
8. NS
9. Extracellular matrix organization

Network analysis of down-regulated proteins in H_2O_2-treated ARPE-19 cells. Protein interaction networks were download from STRING database and visualised with Cytoscape. The network was clustered according to the strength of interaction (combined score from STRING) and each cluster was analysed for functional enrichment of biological processes. Color of the nodes is relative to the log2 fold change of protein expression H_2O_2 vs Ctr (non-treated). The width of the edges is proportional to the strength of the interaction. Nine clusters showed significant association with specific biological process, which are reported below the figure with the corresponding number.

Supplementary figure S4

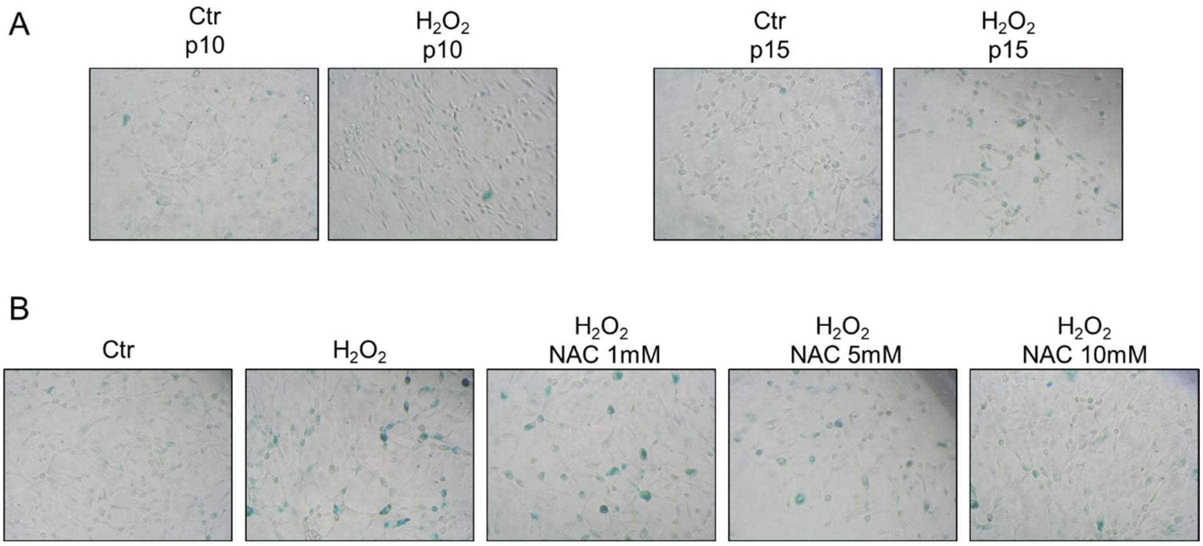

Figure S4. Chronic sub-lethal oxidative insult induces senescence in ARPE-19 cells. A) Ctr and H2O2 treated cells at different passage number were seeded in triplicate in H2O2-free medium and grown in for 72h before processing. B) Ctr and H2O2 treated cells at passage 18 were seeded in triplicate in H2O2-free medium either in the presence or in the absence of N-Acetyl cysteine (NAC) at the indicated concentrations. Cells were grown for 72h before performing SA-β-Gal activity assay. Senescent cells are stained in blue, magnification 20X.
